# Determinants of publication likelihood and timeliness for clinical studies

**DOI:** 10.1017/cts.2025.10201

**Published:** 2025-12-04

**Authors:** Haoyuan Wang, Le Li, Chuan Hong, Rui Yang, Karen Chiswell, Sara B. Calvert, Lesley Curtis, Ali B. Abbasi, Scott Michael Palmer, Adrian F. Hernandez, Frank W. Rockhold, Christopher Lindsell

**Affiliations:** 1 Department of Biostatistics and Bioinformatics, Duke University, Durham, NC, USA; 2 University of Texas at Austin, Austin, TX, USA; 3 Duke Clinical Research Institute, Duke University, Durham, NC, USA; 4 Department of Biomedical Informatics, Yong Loo Lin School of Medicine, National University of Singapore, Singapore, Singapore; 5 Department of Medicine, Duke University, Durham, NC, USA; 6 Department of Surgery, University of California, San Francisco, CA, USA

**Keywords:** Survival analysis, interventional trials, clinical trial feature, predictive modeling, dissemination

## Abstract

**Introduction:**

Timely dissemination of clinical trial results is essential to advance knowledge, guide practice, and improve outcomes, yet many trials remain unpublished, limiting impact. We examine what drives publication and timelines across three major clinical domains.

**Methods:**

We analyzed study design and factors associated with dissemination of interventional trials, focusing on cardiovascular disease (CVD), cancer, and COVID-19. A total of 10,785 trials (CVD: 5929; cancer: 4210; COVID-19: 646) were linked to PubMed publications using National Clinical Trial identifiers. Study design, operational, and transparency-related features were assessed as predictors of time to publication, defined as the interval from study completion to first publication, using Cox proportional hazards model.

**Results:**

COVID-19 trials had the highest publication rate (49.6%), followed by CVD (42.3%) and cancer (32.9%), likely reflecting pandemic-related prioritization. Faster publication was associated with larger enrollment, more sites, result posting, randomization, DMC presence, and higher blinding levels (all *p* < 0.05). Slower publication was linked to supportive care or diagnostic trials (CVD), basic science (cancer), and later COVID-19 trial completion. In subgroups, U.S. facility presence (CVD) and phase 3 design (cancer) predicted faster publication, while healthy volunteer inclusion (CVD) predicted slower publication. Among DMC trials, more secondary outcomes were linked to faster publication across all disease areas.

**Conclusions:**

Key study design and operational factors consistently predict whether and when trials are published. Strengthening methodological rigor, result reporting, and multi-site collaboration may accelerate timely dissemination into peer-reviewed literature.

## Introduction

The dissemination of clinical research findings is vital for advancing medical knowledge, guiding evidence-based practices, and improving patient outcomes. Despite the increasing number of clinical trials conducted globally, a significant proportion of these studies fail to progress to publication [1]. This limits their impact on the scientific community and healthcare decision-making, highlighting the importance of identifying and addressing barriers to the dissemination of clinical trial findings. Such selective availability of evidence increases the risk of clinicians making decisions based on incomplete or skewed data, with potential harm to patients and public health. Recognizing these dangers underscores the importance of understanding the factors that influence publication likelihood to ensure that valuable research outcomes remain accessible and inform clinical practice and policy [2].

Several barriers to publication have been documented, including limitations in study design, lack of transparency, and operational challenges [2]. Single-site trials, for instance, are often perceived as less rigorous or generalizable compared to multi-center randomized controlled trials, leading to less desirability for publication [3]. Additionally, design features like the presence of data monitoring committees (DMCs) and masking protocols are associated with increased credibility and higher publication rates, underscoring the importance of study rigor in promoting research dissemination [4].

Clinical trial registries and machine learning tools, such as TrialEnroll [5] and LIFTED [6] have been used to optimize operational efficiencies, predict enrollment success and trial outcomes [7, 8, 9, 10, 11]. However, gaps in timely result reporting and publication persist.

This study builds on prior efforts by examining what drives publication and timelines across three major clinical domains: cardiovascular disease (CVD), cancer, and COVID-19. These areas reflect different research contexts: chronic high-burden (CVD), complex innovation-driven (cancer), and urgent public health response (COVID-19). Using a large ClinicalTrials.gov dataset, we analyze patterns and predictors of publication likelihood and speed, with a focus on design, operations, and transparency.

## Materials and methods

### Cohort definition & inclusion/exclusion criteria

This observational cohort study used data from ClinicalTrials.gov and PubMed to examine factors associated with whether and when interventional clinical trials are published. The study population consisted of interventional trials registered on ClinicalTrials.gov. Data for interventional trials were programmatically extracted from ClinicalTrials.gov using structured fields for study characteristics, operational details, masking protocols, and outcome measures. Trials were identified based on their disease category using disease-specific terms from the conditions and browse_conditions tables. Disease-specific terms were grouped into the three target categories (CVD, cancer, and COVID-19) by mapping condition keywords and MeSH terms to broader disease domains using a standardized codebook developed through clinical expert review and prior published taxonomies [12].

Eligible trials were interventional studies of CVD, cancer, or COVID-19 registered on or after January 1, 2010 and completed before January 1, 2023. Trials were required to be available in the AACT database with a National Clinical Trial (NCT) number available.

### Outcomes ascertainment

To link trials with corresponding publications, PubMed was searched for articles that contained the trial’s NCT identifier in their abstract, keywords, or body text. An automated algorithm facilitated this process, ensuring precise matching and minimizing errors associated with manual linkage (Appendix A of Supplementary Materials) [12]. For trials without a direct match, additional metadata, including study title, principal investigator (PI) name, and publication year, were used to perform supplementary searches. This approach maximized linkage completeness while maintaining accuracy. Multiple publications per trial were recorded to capture the full publication history.

The primary outcome was the time from a trial’s primary completion date (index date) to its first linked publication. Publications that appeared before the primary completion date (typically protocol or design papers) were not counted toward this outcome. Trials with at least one eligible publication before January 1, 2025, were considered to have an observed event; those without were administratively censored on January 1, 2025. We note that posting results to ClinicalTrials.gov is distinct from publication in a peer-reviewed journal; the primary outcome for this study refers specifically to the latter.

To address the potential misclassification of linked publications that may not represent primary results (e.g., protocol papers, systematic reviews, or commentaries), we conducted a targeted manual check of a random subset of linked articles. For a sample of 100 linked publications across CVD, cancer, and COVID-19, we reviewed abstracts to confirm that the publication reported primary or secondary trial results. This spot check indicated that the large majority (>90%) were indeed primary result papers, suggesting limited risk of systematic misclassification. We acknowledge that some protocol or secondary analyses may remain in the linked set, but their proportion is expected to be small.

In instances where the first linked publication date preceded the trial’s recorded primary completion date – which typically indicates a protocol paper or interim report – these publications were excluded from the primary time-to-event outcome to ensure that only result reports contributed to the time-to-publication measure. Trials with only pre-completion publications but no post-completion result publications were considered unpublished for the main outcome. Publications that appeared before trial completion were excluded. Trials with only pre-completion publications and no post-completion result publications were considered unpublished for the main outcome.

### Statistical analysis

#### Study characteristics and comparison

Categorical variables are presented as frequencies and percentages, and continuous variables are summarized using means with standard deviations (SDs) and medians where appropriate. The descriptive variables included study design characteristics such as the presence of a DMC, whether the study had a U.S.-based facility, whether it was multi-site trial or not, masking level (none, single, double, triple, or quadruple), intervention model (parallel, crossover, single-group, or other), allocation (randomized or non-randomized), primary purpose, inclusion of healthy volunteers, gender eligibility, inclusion of adults, children, or older adults, minimum age, and whether the study provided expanded access. Operational characteristics included funding source (Government, Private, or Other), total number of facilities, classification of arms (number of arms = 1, 2, or >2), enrollment size, number of primary and secondary outcomes to be measured, trial duration measures (e.g., months to completion and primary completion), and whether the study was classified as multiple records. For each disease type (i.e., CVD or Cancer or COVID-19), variables with more than 30% missing data were excluded from the analysis (Appendix B of Supplementary Materials). For the remaining variables, missingness was addressed through complete case analysis. COVID-19 analyses also include the calendar year of trial completion date as one additional predictor, to account for rapid changes in research context and urgency over the course of the pandemic.

The outcome variable, “Publication,” represents whether a corresponding PubMed publication was observed. Bivariate analyses compared trials with and without a publication. Chi-squared tests or Fisher’s exact tests were used to compare categorical variables, while t-tests were employed for continuous variables, depending on their distribution. Kaplan-Meier curves and log-rank tests were used to compare time-to-event outcomes (e.g., time to the publication) across key predictor groups.

To examine factors associated with publication likelihood, the Cox Proportional Hazards (Cox PH) model was applied. The PH assumption was tested using Schoenfeld residuals, and stratification was used if the assumption was violated for any variable. All study design and operational characteristics described above were included as covariates in the models. For COVID-19 trials, the calendar year of trial completion was also included to account for changes in the research context during the pandemic. Categorical variables were regrouped for interpretability and converted to factors. Reference categories generally reflected the most common or policy-relevant level; for example, “Government” (funding source), “Early Phase” (phase), “Treatment” (primary purpose), “Parallel” (intervention model), “None” (masking), “Randomized” (allocation), and “all” (gender eligibility).

## Results

### Study characteristics and comparison

#### Overall study characteristics

As shown in Table 1, studies commonly had a DMC, with proportions ranging from about 45% for CVD trials to over 59% for COVID-19 studies. Most trials were funded by non-governmental sponsors, with “Other” or private sources – including individual and industry funding – accounting for the vast majority across all disease areas. Enrollment sizes varied widely, with cancer trials averaging 120 participants (median 43) and COVID-19 studies averaging 4344 participants (median 120). Most studies focused on treatment and used parallel or single-group designs. Randomization was used in most trials: about 90% for CVD and in COVID-19 trials. Blinding levels ranged widely from none to quadruple; open-label designs remained common, especially in cancer trials. Eligibility was generally open to all genders and age groups, though inclusion of children was rare. The median number of secondary outcomes typically ranged from about four to five. Expanded access programs were uncommon in all three areas. See Table S1 of Supplementary Materials for variable details.

**Table 1.** Descriptive characteristics of clinical studies across three types of studies

Variables			Value		
	Category		CVD	CANCER	COVID
*N*			6617	4525	728
Have publication, no. (%)	No		3821 (57.7)	3035 (67.1)	367 (50.4)
	Yes		2796 (42.3)	1490 (32.9)	361 (49.6)
Has DMC, no. (%)	No		3643 (55.1)	2112 (46.7)	296 (40.7)
	Yes		2974 ( 44.9)	2413 (53.3)	432 (59.3)
Source, no. (%)	Government	Fed	72 (1.1)	4 (0.1)	1 (0.1)
		NIH	9 (0.1)	104 (2.3)	4 (0.5)
		Other Gov	157 (2.4)	29 (0.6)	48 (6.6)
	Private	Individual	6 (0.1)	0 (0)	0 (0)
		Industry	1388 (21.0)	1831 (40.5)	223 (30.6)
		Network	38 (0.6)	103 (2.3)	13 (1.8)
	Other	Other	4942 (74.7)	2438 (53.9)	436 (59.9)
		Unknown	5 (0.1)	16 (0.4)	3 (0.4)
Phase, no. (%)	Early phase			2104 (46.5)	
	Phase 2			1765 (39.0)	
	Phase 3			506 (11.2)	
	Phase 4			150 (3.3)	
Actual duration, mean (SD)			27.83 (21.05)	40.04 (23.48)	6.18 (4.38)
Results reported, no. (%)	No		4962 (75.0)	2752 (60.8)	530 (72.8)
	Yes		1655 (25.0)	1773 (39.2)	198 (27.2)
Enrollment, mean (SD)			3898.02 (248182.88)	119.66 (416.12)	4344.03 (69,930.09)
Gender, no. (%)	All		6165 (93.2)	3596 (79.5)	717 (98.5)
	Female		225 (3.4)	576 (12.7)	6 (0.8)
	Male		227 (3.4)	353 (7.8)	5 (0.7)
Expanded access, no. (%)	No		6609 (99.9)	4477 (98.9)	724 (99.5)
	Yes		8 (0.1)	48 (1.1)	4 (0.5)
Number of facilities, mean (SD)			10.25 (49.63)	15.15 (43.81)	8.02 (20.80)
Has U.S. facility, no. (%)	No		4363 (65.9)	2035 (45.0)	533 (73.2)
	Yes		2254 (34.1)	2490 (55.0)	195 (26.8)
Single facility, no. (%)	No		2095 (31.7)	2489 (55.0)	289 (39.7)
	Yes		4522 (68.3)	2036 (45.0)	439 (60.3)
Primary purpose, no. (%)	Treatment		4250 (64.2)	3905 (86.3)	465 (63.9)
	Supportive care		351 (5.3)	138 (3.0)	26 (3.6)
	Prevention		951 (14.4)	160 (3.5)	154 (21.2)
	Diagnostic		238 (3.6)	148 (3.3)	17 (2.3)
	Basic science		290 (4.4)	56 (1.2)	10 (1.4)
	Health services research		222 (3.4)	20 (0.4)	15 (2.1)
	Other		315 (4.8)	98 (2.2)	41 (5.6)
Number of arms, mean (SD)			2.40 (1.13)	1.91 (1.69)	2.67 (1.68)
Classification of arms, no. (%)	Number of arms = 1		67 (1.0)	2312 (51.1)	
	Number of arms = 2		5091 (76.9)	1474 (32.6)	537 (73.8)
	Number of arms > 2		1459 (22.0)	739 (16.3)	191 (26.2)
Intervention model, no. (%)	Parallel		5341 (80.7)	1710 (37.8)	646 (88.7)
	Crossover		758 (11.5)	76 (1.7)	18 (2.5)
	Single group		305 (4.6)	2517 (55.6)	10 (1.4)
	Other		213 (3.2)	222 (4.9)	54 (7.4)
Allocation, no. (%)	Non randomized		622 (9.4)		73 (10.0)
	Randomized		5995 (90.6)		655 (90.0)
Healthy volunteers, no. (%)	No		5422 (81.9)	4375 (96.7)	498 (68.4)
	Yes		1195 (18.1)	150 (3.3)	230 (31.6)
Months to completion date, mean (SD)			27.83 (20.83)	39.92 (23.19)	6.53 (4.33)
Months to study first submitted date, mean (SD)			4.75 (17.81)	1.51 (14.43)	1.17 (5.68)
Months to last updated submitted date, mean (SD)			51.07 (32.64)	72.44 (34.32)	19.42 (13.02)
Months to study first posted date, mean (SD)			5.84 (18.53)	2.35 (15.14)	1.48 (5.78)
Number of primary outcomes to measure, mean (SD)			1.67 (2.12)	1.71 (2.11)	2.17 (3.01)
Number of secondary outcomes to measure, mean (SD)			5.65 (6.96)	5.39 (5.47)	8.14 (9.09)
Blinding, no. (%)	None		2610 (39.4)	3948 (87.2)	295 (40.5)
	Single		1569 (23.7)	117 (2.6)	84 (11.5)
	Double		940 (14.2)	164 (3.6)	133 (18.3)
	Triple		597 (9.0)	110 (2.4)	74 (10.2)
	Quadruple		901 (13.6)	186 (4.1)	142 (19.5)
Minimum age, mean (SD)			8083.52 (4149.36)	6939.24 (2730.66)	6950.41 (2785.38)
Include adults, no. (%)	No		235 (3.6)	53 (1.2)	15 (2.1)
	Yes		6382 (96.4)	4472 (98.8)	713 (97.9)
Include children, no. (%)	No		6287 (95.0)	4348 (96.1)	681 (93.5)
	Yes		330 (5.0)	177 (3.9)	47 (6.5)
Include older adults, no. (%)	No		731 (11.0)	179 (4.0)	86 (11.8)
	Yes		5886 (89.0)	4346 (96.0)	642 (88.2)
Is multiple, no. (%)	No		5266 (79.6)	3995 (88.3)	569 (78.2)
	Yes		1351 (20.4)	530 (11.7)	159 (21.8)
Results count, mean (SD)			0.42 (0.49)	0.33 (0.47)	0.50 (0.50)
Year group, no. (%)	1				690 (94.8)
	2				38 (5.2)
Enrollment, median (IQR)			79 (162)	43 (81)	120 (283.25)
Actual duration, median (IQR)			24 (26)	36 (28)	6 (6)
Number of primary outcomes to measure, median (IQR)			1 (1)	1 (1)	1 (1)
Number of secondary outcomes to measure, median (IQR)			4 (5)	4 (5)	6 (8)

#### Variation across disease areas

CVD trials (*N* = 6617) were notable for larger average enrollments (mean ∼ 3898, median ∼ 79) and moderate trial duration (mean ∼ 28, median ∼ 24 months). They were more likely to be conducted at a single site (68.3%) and less likely to include a U.S. facility (34.1%) compared to cancer trials. Cancer trials (*N* = 4525) tended to run longer (mean duration ∼ 40 months) but enrolled far fewer participants (mean ∼ 120, median 36), consistent with their frequent focus on smaller, tightly controlled early-phase studies. Over half of cancer trials were single-arm (51.1%) or two-arm (32.6%), and nearly 87% used no blinding, indicating open-label designs were dominant. COVID-19 studies were also more likely to use parallel designs (88.7%) and had relatively high rates of randomization (90%).

U.S. facility inclusion was highest for cancer trials (55%) and lowest for COVID-19 studies (26.8%). Cancer studies continued to have the highest proportion reporting results (39.2) in ClinicalTrials.gov, compared to CVD (25.0%) and COVID-19 (27.2%). Blinding patterns varied substantially: most cancer studies were open-label, while COVID-19 studies more frequently used higher-level masking than CVD trials. Minimum participant age and use of healthy volunteers also varied, with COVID-19 trials more frequently including healthy volunteers (31.6%) than CVD (18.1%) or cancer (3.3%) studies.

### Factors associated with publication

Overall, publication was more common for COVID-19 trials (49.6%) than for CVD (42.3%) or cancer (32.9%). As shown in Table 2, across all three disease areas, published trials consistently showed patterns of greater operational scale, stronger oversight, and more robust design features compared to unpublished trials. Published trials were more likely to have a DMC, to report results, to be randomized, and to use a parallel intervention model. They also tended to involve multiple facilities, rather than single sites, and to apply higher levels of masking. Published studies generally reported more secondary outcomes and showed faster submission and posting timelines. While the mean enrollment was higher for published cancer trials than unpublished cancer trials, a similar difference was not observed for CVD or COVID-19.

**Table 2.** Comparison of study characteristics between clinical trials with and without a linked PubMed publication

**A. Study characteristics for CVD trials**					
Variables	Category		Publication = 0	Publication = 1	*P*-value
*N*			3821	2796	
Has DMC, no. (%)	No		2309 (60.4)	1334 (47.7)	<0.001***
	Yes		1512 (39.6)	1462 (52.3)	
**Source, no. (%)**	Government	Fed	51 (1.3)	21 (0.8)	0.321
		NIH	5 (0.1)	4 (0.1)	
		Other Gov	94 (2.5)	63 (2.3)	
	Private	Individual	4 (0.1)	2 (0.1)	
		Industry	811 (21.2)	577 (20.6)	
		Network	19 (0.5)	19 (0.7)	
	Other	Other	2833 ( 74.1)	2109 (75.4)	
		Unknown	4 (0.1)	1 (0.0)	
Actual duration, mean (SD)			26.23 (21.87)	30.02 (19.68)	<0.001***
Results reported, no. (%)	No		2996 (78.4)	1966 (70.3)	<0.001***
	Yes		825 (21.6)	830 (29.7)	
Enrollment, mean (SD)			5809.20 (326142.44)	1286.21 (20,272.05)	0.464
Gender, no. (%)	All		3509 (91.8)	2656 (95.0)	<0.001***
	Female		143 (3.7)	82 (2.9)	
	Male		169 (4.4)	58 (2.1)	
Expanded access, no. (%)	No		3818 (99.9)	2791 (99.8)	0.423
	Yes		3 (0.1)	5 (0.2)	
Number of facilities, mean (SD)			4.56 (16.11)	18.02 (73.29)	<0.001***
Has U.S. facility, no. (%)	No		2559 (67.0)	1804 (64.5)	0.04*
	Yes		1262 (33.0)	992 (35.5)	
Single facility, no. (%)	No		1002 (26.2)	1093 (39.1)	<0.001***
	Yes		2819 (73.8)	1703 (60.9)	
Primary purpose, no. (%)	Treatment		2401 (62.8)	1849 (66.1)	<0.001***
	Supportive care		221 (5.8)	130 (4.6)	
	Prevention		540 (14.1)	411 (14.7)	
	Diagnostic		167 (4.4)	71 (2.5)	
	Basic science		190 (5.0)	100 (3.6)	
	Health services research		112 (2.9)	110 (3.9)	
	Other		190 (5.0)	125 (4.5)	
Number of arms, mean (SD)			2.40 (1.15)	2.39 (1.11)	0.722
Classification of arms, no. (%)	Number of arms = 1		54 (1.4)	13 (0.5)	0.001***
	Number of arms = 2		2914 (76.3)	2177 (77.9)	
	Number of arms > 2		853 (22.3)	606 (21.7)	
Intervention model, no. (%)	Parallel		2997 (78.4)	2344 (83.8)	<0.001***
	Crossover		488 (12.8)	270 (9.7)	
	Single group		214 (5.6)	91 (3.3)	
	Other		122 (3.2)	91 (3.3)	
Allocation, no. (%)	Non randomized		457 (12.0)	165 (5.9)	<0.001***
	Randomized		3364 (88.0)	2631 (94.1)	
Healthy volunteers, no. (%)	No		3014 (78.9)	2408 (86.1)	<0.001***
	Yes		807 (21.1)	388 (13.9)	
Months to completion date, mean (SD)			26.24 (21.63)	30.00 (19.48)	<0.001***
Months to study first submitted date, mean (SD)			6.34 (19.99)	2.58 (14.03)	<0.001***
Months to last updated submitted date, mean (SD)			49.18 (33.50)	53.64 (31.24)	<0.001***
Months to study first posted date, mean (SD)			7.59 (20.81)	3.45 (14.53)	<0.001***
Number of primary outcomes to measure, mean (SD)			1.72 (2.24)	1.61 (1.94)	0.056
Number of secondary outcomes to measure, mean (SD)			4.85 (5.21)	6.75 (8.68)	<0.001***
Blinding, no. (%)	None		1614 (42.2)	996 (35.6)	<0.001***
	Single		883 (23.1)	686 (24.5)	
	Double		549 (14.4)	391 (14.0)	
	Triple		344 (9.0)	253 (9.0)	
	Quadruple		431 (11.3)	470 (16.8)	
Minimum age, mean (SD)			8046.82 (4110.49)	8133.69 (4202.12)	0.4
Include adults, no. (%)	No		139 (3.6)	96 (3.4)	0.707
	Yes		3682 (96.4)	2700 (96.6)	
Include children, no. (%)	No		3618 (94.7)	2669 (95.5)	0.172
	Yes		203 (5.3)	127 (4.5)	
Include older adults, no. (%)	No		510 (13.3)	221 (7.9)	<0.001***
	Yes		3311 (86.7)	2575 (92.1)	
Is multiple, no. (%)	No		3636 (95.2)	1630 (58.3)	<0.001***
	Yes		185 (4.8)	1166 (41.7)	
Results count, mean (SD)			0.00 (0.00)	1.00 (0.00)	<0.001***
Enrollment, median (IQR)			60 (99)	114 (265)	<0.001***
Actual duration, median (IQR)			21 (26)	27 (26)	<0.001***
Number of primary outcomes to measure, median (IQR)			1 (1)	1 (0)	0.01*
Number of secondary outcomes to measure, median (IQR)			3 (4)	5 (6)	<0.001***

B. Study characteristics for cancer trials					
*N*			3035	1490	
Has DMC, no. (%)	No		1457 (48.0)	655 (44.0)	0.011*
	Yes		1578 (52.0)	835 (56.0)	
**Source, no. (%)**	Government	Fed	3 (0.1)	1 (0.1)	0.02*
		NIH	69 (2.3)	35 (2.3)	
		Other Gov	18 (0.6)	11 (0.7)	
	Private	Individual	0 (0)	0 (0)	
		Industry	1174 (38.7)	657 (44.1)	
		Network	68 (2.2)	35 (2.3)	
	Other	Other	1694 (55.8)	744 (49.9)	
		Unknown	9 (0.3)	7 (0.5)	
Phase, no. (%)	Early Phase		1533 (50.5)	571 (38.3)	<0.001***
	Phase 2		1163 (38.3)	602 (40.4)	
	Phase 3		238 (7.8)	268 (18.0)	
	Phase 4		101 (3.3)	49 (3.3)	
Actual duration, mean (SD)			40.44 (24.47)	39.23 (21.31)	0.105
Results reported, no. (%)	No		1941 (64.0)	811 (54.4)	<0.001***
	Yes		1094 (36.0)	679 (45.6)	
Enrollment, mean (SD)			85.97 (237.56)	188.30 (635.66)	<0.001***
Gender, no. (%)	All		2413 (79.5)	1183 (79.4)	0.459
	Female		377 (12.4)	199 (13.4)	
	Male		245 (8.1)	108 (7.2)	
Expanded access, no. (%)	No		3011 (99.2)	1466 (98.4)	0.018*
	Yes		24 (0.8)	24 (1.6)	
Number of facilities, mean (SD)			10.23 (30.35)	25.17 (61.69)	<0.001***
Has U.S. facility, no. (%)	No		1305 (43.0)	730 (49.0)	<0.001***
	Yes		1730 (57.0)	760 (51.0)	
Single facility, no. (%)	No		1557 (51.3)	932 (62.6)	<0.001***
	Yes		1478 (48.7)	558 (37.4)	
Primary purpose, no. (%)	Treatment		2594 (85.5)	1311 (88.0)	<0.001***
	Supportive care		100 (3.3)	38 (2.6)	
	Prevention		98 (3.2)	62 (4.2)	
	Diagnostic		108 (3.6)	40 (2.7)	
	Basic science		52 (1.7)	4 (0.3)	
	Health services research		12 (0.4)	8 (0.5)	
	Other		71 (2.3)	27 (1.8)	
Number of arms, mean (SD)			1.83 (1.46)	2.06 (2.07)	<0.001***
Classification of arms, No. (%)	Number of arms = 1		1657 (54.6)	655 (44.0)	<0.001***
	Number of arms = 2		893 (29.4)	581 (39.0)	
	Number of arms > 2		485 (16.0)	254 (17.0)	
Intervention Model, no. (%)	Parallel		1047 (34.5)	663 (44.5)	<0.001***
	Crossover		56 (1.8)	20 (1.3)	
	Single group		1786 (58.8)	731 (49.1)	
	Other		146 (4.8)	76 (5.1)	
Healthy volunteers, no. (%)	No		2921 (96.2)	1454 (97.6)	0.023*
	Yes		114 (3.8)	36 (2.4)	
Months to completion date, mean (SD)			40.31 (24.16)	39.12 (21.05)	0.106
Months to study first submitted date, mean (SD)			1.96 (15.22)	0.61 (12.64)	0.003**
Months to last updated submitted date, mean (SD)			71.94 (35.21)	73.45 (32.42)	0.164
Months to study first posted date, mean (SD)			2.89 (16.10)	1.24 (12.89)	0.001***
Number of primary outcomes to measure, mean (SD)			1.75 (2.30)	1.62 (1.67)	0.045*
Number of secondary outcomes to measure, mean (SD)			4.89 (4.88)	6.40 (6.39)	<0.001***
Blinding, no. (%)	None		2695 (88.8)	1253 (84.1)	<0.001***
	Single		79 (2.6)	38 (2.6)	
	Double		101 (3.3)	63 (4.2)	
	Triple		63 (2.1)	47 (3.2)	
	Quadruple		97 (3.2)	89 (6.0)	
Minimum age, mean (SD)			6948.04 (2758.97)	6921.30 (2672.89)	0.757
Include adults, no. (%)	No		34 (1.1)	19 (1.3)	0.758
	Yes		3001 (98.9)	1471 (98.7)	
Include children, no. (%)	No		2912 (95.9)	1436 (96.4)	0.537
	Yes		123 (4.1)	54 (3.6)	
Include older adults, no. (%)	No		129 (4.3)	50 (3.4)	0.171
	Yes		2906 (95.7)	1440 (96.6)	
Is multiple, no. (%)	No		2959 (97.5)	1036 (69.5)	<0.001***
	Yes		76 (2.5)	454 (30.5)	
Results count, mean (SD)			0.00 (0.00)	1.00 (0.00)	<0.001***
Enrollment, median (IQR)			37 (56)	66 (141)	<0.001***
Actual duration, median (IQR)			36 (30)	36 (27)	0.738
Number of primary outcomes to measure, median (IQR)			1 (1)	1 (1)	0.738
Number of secondary outcomes to measure, median (IQR)			4 (4)	5 (5)	<0.001***

C. Study characteristics for COVID trials					
*N*			367	361	
Has DMC, no. (%)	No		173 (47.1)	123 (34.1)	<0.001***
	Yes		194 (52.9)	238 (65.9)	
**Source, no. (%)**	Government	Fed	0 (0.0)	1 (0.3)	0.617
		NIH	3 (0.8)	1 (0.3)	
		Other Gov	26 (7.1)	22 (6.1)	
	Private	Individual	0 (0)	0 (0)	
		Industry	120 (32.7)	103 (28.5)	
		Network	6 (1.6)	7 (1.9)	
	Other	Other	211 (57.5)	225 (62.3)	
		Unknown	1 (0.3)	2 (0.6)	
Results reported, no. (%)	No		289 (78.7)	241 (66.8)	<0.001***
	Yes		78 (21.3)	120 (33.2)	
Enrollment, mean (SD)			6587.07 (96,690.92)	2063.71 (18,979.64)	0.383
Gender, no. (%)	All		361 (98.4)	356 (98.6)	0.911
	Female		3 (0.8)	3 (0.8)	
	Male		3 (0.8)	2 ( 0.6)	
Expanded access, no. (%)	No		367 (100.0)	357 (98.9)	0.128
	Yes		0 (0.0)	4 (1.1)	
Number of facilities, mean (SD)			4.68 (12.12)	11.41 (26.48)	<0.001***
Has U.S. facility, no. (%)	No		286 (77.9)	247 (68.4)	0.005**
	Yes		81 (22.1)	114 (31.6)	
Single facility, no. (%)	No		130 (35.4)	159 (44.0)	0.021*
	Yes		237 (64.6)	202 (56.0)	
Primary purpose, no. (%)	Treatment		227 (61.9)	238 (65.9)	0.208
	Supportive care		13 (3.5)	13 (3.6)	
	Prevention		76 (20.7)	78 (21.6)	
	Diagnostic		14 (3.8)	3 (0.8)	
	Basic science		6 (1.6)	4 (1.1)	
	Health services research		8 (2.2)	7 (1.9)	
	Other		23 (6.3)	18 (5.0)	
Number of arms, mean (SD)			2.66 (1.67)	2.68 (1.69)	0.894
Classification of arms, no. (%)	Number of arms = 2		272 (74.1)	265 (73.4)	0.894
Number of arms > 2			95 (25.9)	96 (26.6)	
Intervention model, no. (%)	Parallel		315 (85.8)	331 (91.7)	0.073
	Crossover		13 (3.5)	5 (1.4)	
	Single group		6 (1.6)	4 (1.1)	
	Other		33 (9.0)	21 (5.8)	
Allocation, no. (%)	Non randomized		51 (13.9)	22 (6.1)	0.001***
	Randomized		316 (86.1)	339 (93.9)	
Healthy volunteers, no. (%)	No		242 (65.9)	256 (70.9)	0.173
	Yes		125 (34.1)	105 (29.1)	
Months to completion date, mean (SD)			6.11 (4.07)	6.96 (4.54)	0.008**
Months to study first submitted date, mean (SD)			2.23 (7.19)	0.10 (3.22)	<0.001***
Months to last updated submitted date, mean (SD)			18.31 (12.21)	20.56 (13.72)	0.02*
Months to study first posted date, mean (SD)			2.59 (7.29)	0.36 (3.29)	<0.001***
Number of primary outcomes to measure, mean (SD)			2.17 (3.15)	2.17 (2.86)	0.99
Number of secondary outcomes to measure, mean (SD)			7.24 (7.69)	9.06 (10.24)	0.007**
Blinding, no. (%)	None		162 (44.1)	133 (36.8)	0.355
	Single		42 (11.4)	42 (11.6)	
	Double		63 (17.2)	70 (19.4)	
	Triple		35 (9.5)	39 (10.8)	
	Quadruple		65 (17.7)	77 (21.3)	
Minimum age, mean (SD)			6980.55 (2719.27)	6919.77 (2854.47)	0.769
Include adults, no. (%)	No		7 (1.9)	8 (2.2)	0.974
	Yes		360 (98.1)	353 (97.8)	
Include children, no. (%)	No		346 (94.3)	335 (92.8)	0.508
	Yes		21 (5.7)	26 (7.2)	
Include older adults, no. (%)	No		42 (11.4)	44 (12.2)	0.844
	Yes		325 (88.6)	317 (87.8)	
Is multiple, no. (%)	No		353 (96.2)	216 (59.8)	<0.001***
	Yes		14 (3.8)	145 (40.2)	
Results count, mean (SD)			0.00 (0.00)	1.00 (0.00)	<0.001***
Year group, no. (%)	1		333 (90.7)	357 (98.9)	<0.001***
	2		34 (9.3)	4 (1.1)	
Enrollment, median (IQR)			98 (189.5)	160 (368)	<0.001***
Actual duration, median (IQR)			5 (6)	6 (6)	0.453
Number of primary outcomes to measure, median (IQR)			1 (1)	1 (1)	0.453
Number of secondary outcomes to measure, median (IQR)			5 (7)	6 (7)	<0.001***

#### Variation across disease areas

The patterns linking design features to publication varied by condition. For CVD trials, published studies more often had a DMC (52.3% vs. 39.6%), were randomized (94.1% vs. 88%), used parallel designs (83.8% vs. 78.4%), ran longer (30.02 vs. 26.23 months), operated at more sites (18 vs. 5), and measured more secondary outcomes than unpublished CVD studies. In cancer, the strongest differences reflected scale and phase: published trials were more often Phase 3 (18% vs. 8%), enrolled more participants (188 vs. 86), used parallel rather than single-group designs (44.5% vs. 34.5%), ran at more sites (25 vs. 10), and applied higher blinding levels, particularly quadruple blinding (6% vs. 3%). For COVID-19 trials, the clearest variation was operational urgency: published studies more often had a DMC (65.9% vs. 52.9%), used parallel models (97.1% vs. 85.8%), spanned more sites (11 vs. 5), measured more secondary outcomes, and showed faster registration and result reporting than unpublished COVID trials.

#### Variation across U.S. sponsored trials (Supplementary Table S2)

In the subgroup analysis of trials with U.S. sponsors, results showed patterns largely consistent with the overall trials, with only modest differences in magnitude. For CVD trials, published studies more often had a DMC (55.3% vs. 38.4%), were randomized (93.5% vs. 85.2%), and used parallel designs (82.1% vs. 75.8%). They also tended to have longer durations (31.4 vs. 30.1 months) and a greater number of participating sites (23 vs. 5). In cancer trials, the most pronounced differences reflected scale and phase. Published trials were more often Phase 3 (19% vs. 7%), enrolled more participants (181 vs. 86), and used parallel rather than single-group designs (47.2% vs. 33.6%). They also operated at more sites (27 vs. 11) and employed higher levels of blinding, particularly quadruple blinding (6% vs. 3%). For COVID-19 trials, published trials more frequently included a DMC (68.0% vs. 52.2%), used parallel intervention models (91.0% vs. 80.4%), and spanned more study sites (21 vs. 8).

### Kaplan-Meier analysis of publication rates

**Figure 1.**
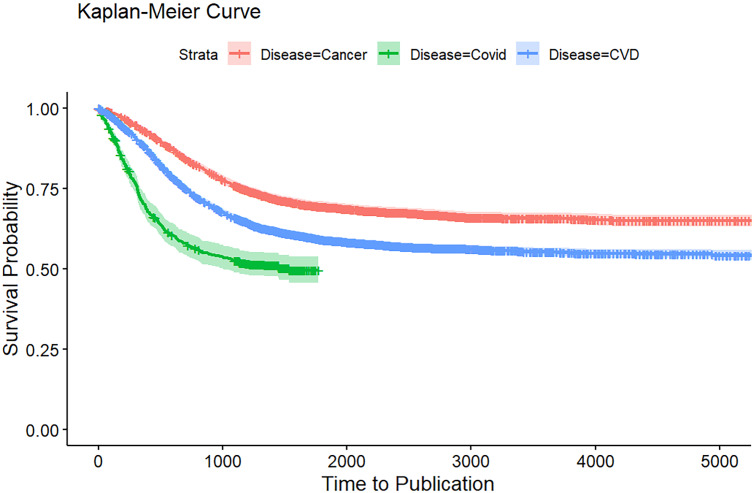
Kaplan-Meier curve for time to publication of CVD, cancer, and COVID-19 clinical studies at ClinicalTrial.gov.

Figure 1 presents the Kaplan–Meier curves comparing time to publication across the three disease areas. COVID-19 trials demonstrated the fastest time to publication, with survival probabilities dropping sharply within the first 1000 days, reflecting rapid dissemination during the pandemic. In contrast, CVD trials showed more moderate publication timelines, while cancer trials had the slowest publication rates, maintaining the highest probability of remaining unpublished over time.

### Factors associated with time to publication

**Figure 2.**
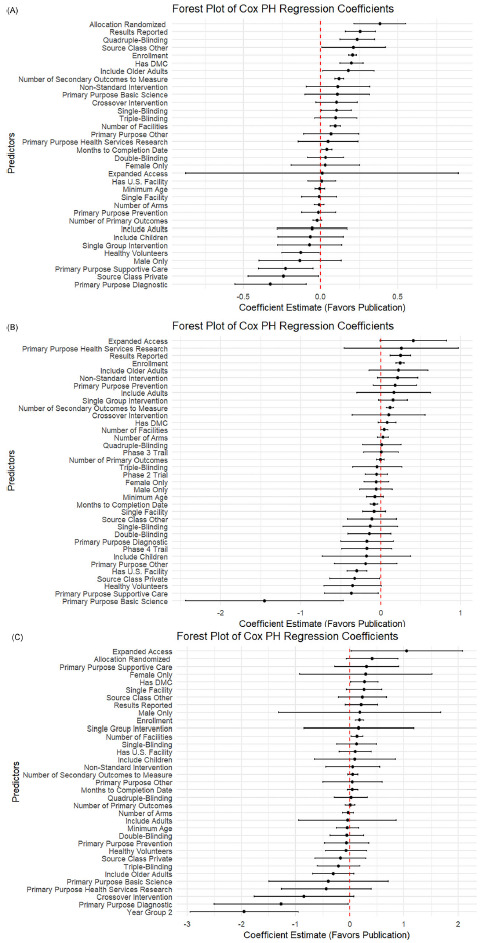
Forest plot of log hazard ratio from Cox PH model for predictors of linked PubMed publication. (A) Forest plot for CVD trials. (B) Forest plot for cancer trials. (C) Forest plot for COVID trials.

As shown in Figure 2, across all three disease areas, the forest plot results showed that trials with greater operational scale and richer data collection tended to publish results more quickly. Larger enrollment sizes, more study sites, and measuring more secondary outcomes were consistent drivers of faster publication for CVD and cancer trials. Indicators of trial rigor (e.g., having a DMC, using robust masking, and reporting results) were significant in some contexts but did not emerge as significant predictors for COVID-19 trials. Offering expanded access was associated with increased publication likelihood for cancer and COVID-19 studies.

#### Variation across disease areas

While some predictors were broadly consistent, the strength and pattern of associations varied by disease. In CVD trials (Figure 2A), multiple operational and design features, such as having a DMC (0.20, *p* < 0.001), randomization (0.38, *p* < 0.001), higher blinding (single: 0.10, *p* = 0.03; quadruple: 0.23, *p* < 0.001), and including older adults (0.18, *p* = 0.03), were strongly linked to faster publication. In contrast, privately funded CVD trials (−0.24, *p* = 0.03) and those focused on supportive care (−0.22, *p* = 0.01) or diagnostics (−0.32, *p* = 0.005) lagged behind.

For cancer trials (Figure 2B), operational scale and transparency stood out more clearly: larger enrollment (0.24, *p* < 0.001), more sites (0.04, *p* = 0.04), more secondary outcomes (0.12, *p* < 0.001), and result reporting (0.25, *p* < 0.001) all favored faster publication. Unique to cancer, basic science trials (−1.45, *p* = 0.003), supportive care (−0.37, *p* = 0.03), and trials with healthy volunteers (−0.35, *p* = 0.05) showed clear delays. COVID-19 trials (Figure 2C) differed: most design features were *not* significant predictors once urgency faded. Instead, larger enrollment (0.18, *p* < 0.001) and expanded access (1.05, *p* = 0.045) drove faster reporting. Crucially, trials registered in later pandemic years were much slower to publish (−1.94, *p* < 0.001).

### Subgroup analysis across trial structure and contextual factors

**Figure 3.**
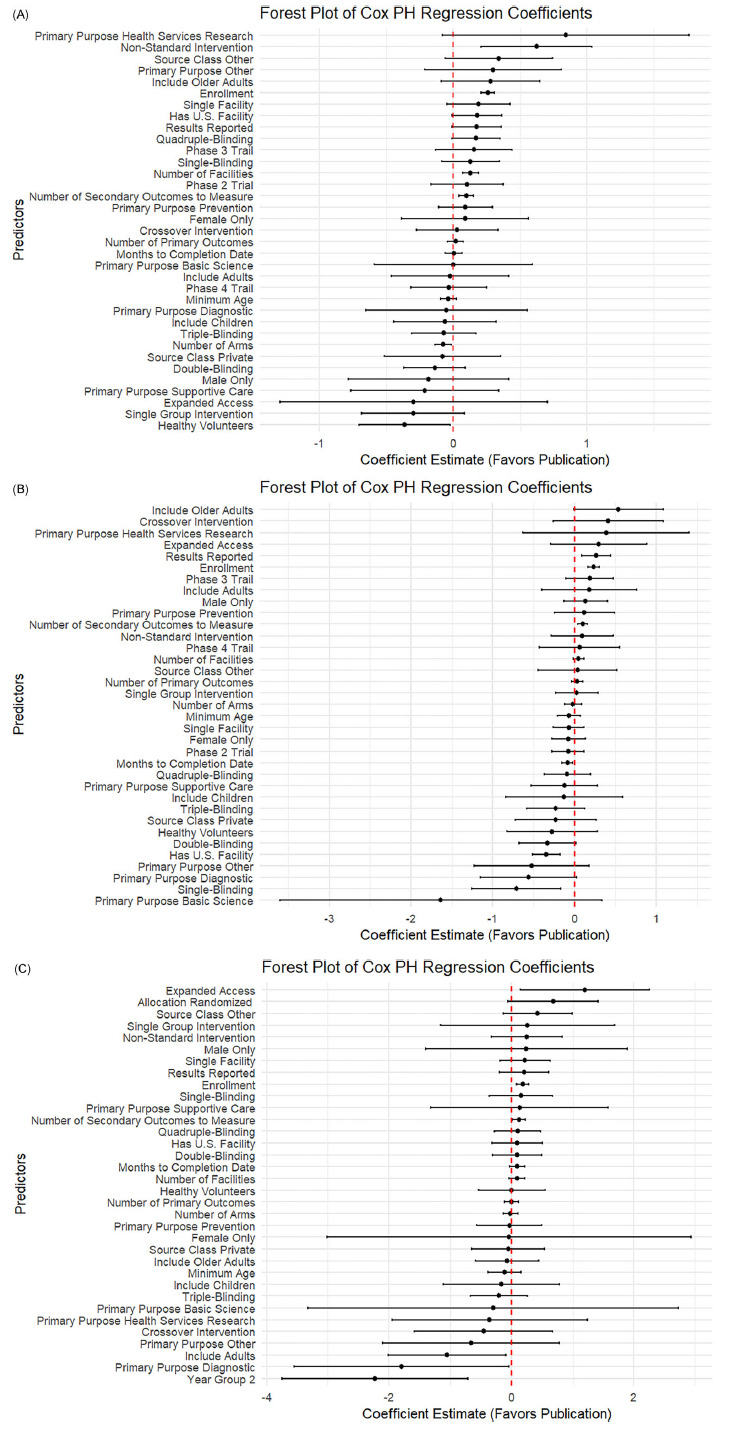
Subgroup analysis among trials with DMC: Forest plot of log hazard ratio from Cox PH model for predictors of linked PubMed publication. (A) Forest plot for CVD trials. (B) Forest plot for cancer trials. (C) Forest plot for COVID trials.

As shown in Figure 3, in rigorously designed trials that included a DMC, a greater number of secondary outcomes was consistently associated with faster publication across all three disease areas: CVD (0.10), cancer (0.09), and COVID-19 (0.12), all with *p < 0.05*. Interestingly, in contrast to the overall analysis, single-arm design was negatively associated with publication in cancer trials (−0.71; *p =* 0.01), suggesting that even among trials with oversight, certain design choices may reduce the likelihood of dissemination.

**Figure 4.**
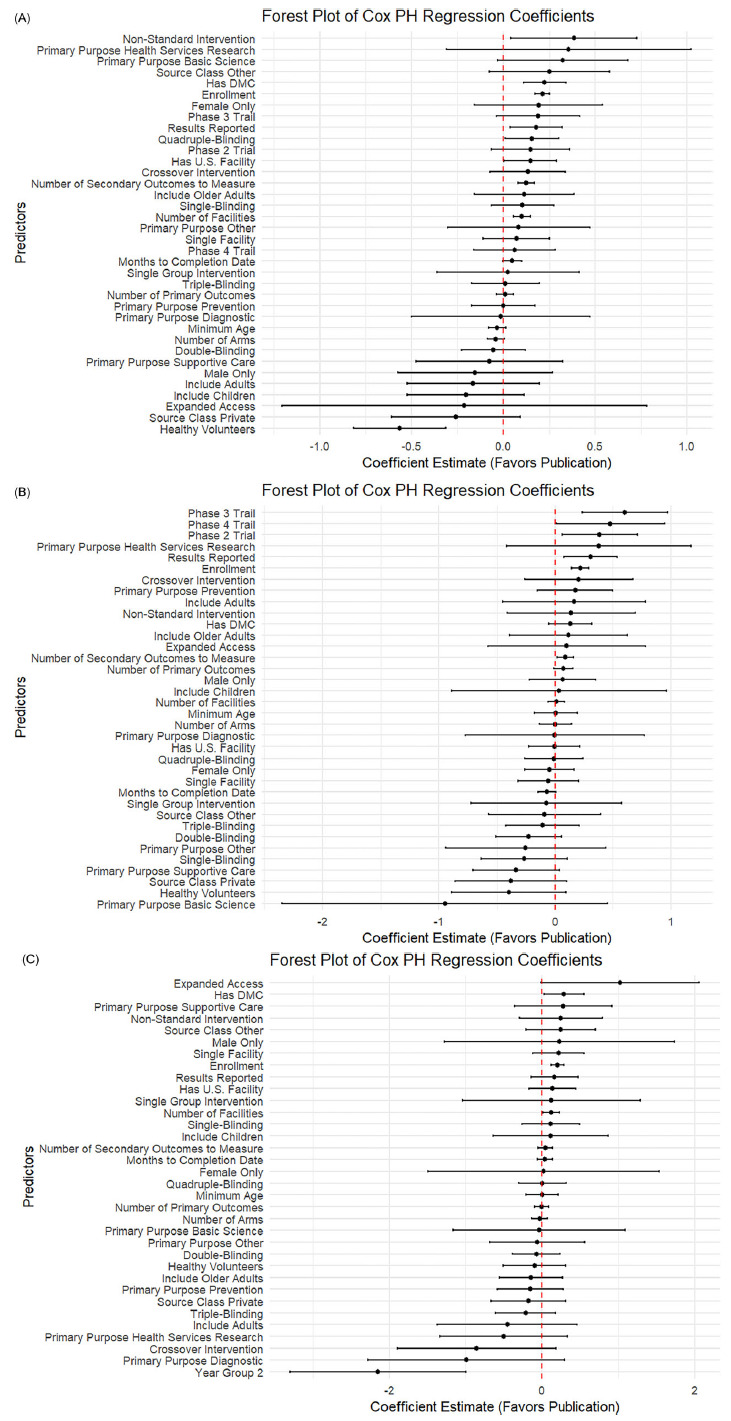
Subgroup analysis among trials with randomized allocation: Forest plot of log hazard ratio from Cox PH model for predictors of linked PubMed publication. (A) Forest plot for CVD trials. (B) Forest plot for cancer trials. (C) Forest plot for COVID trials.

Figure 4 shows results from the subgroup of randomized trials. In cardiovascular studies, the presence of U.S.-based facilities (0.15; *p =* 0.04) and the use of intervention models other than crossover or single-group designs (0.38; *p =* 0.03) were positively associated with publication. In cancer trials, earlier phase designation was linked to more timely publication (0.38 for phase 2, *p* = 0.02; 0.60 for phase 3, *p =* 0.001). In contrast, inclusion of healthy volunteers in cardiovascular trials was associated with reduced publication likelihood (−0.56; *p <* 0.001), possibly reflecting differences in study aims or journal prioritization.

We also examined a subgroup of U.S.-sponsored trials (Supplementary Figure S1). In cardiovascular studies, the presence of a DMC (0.33) and random allocation (0.40) were both positively associated with publication (both *p* < 0.01), as were higher enrollment (0.23), more facilities (0.11), and more secondary outcomes (0.11), all with *p* < 0.001. Conversely, having only a single facility was negatively associated with publication (−0.15; *p* < 0.10). In cancer studies, several factors were negatively associated with publication, including inclusion of healthy volunteers (−0.53), private U.S. sponsors (−0.42), and a primary purpose of basic science (−1.46), all at *p < 0.10*. On the other hand, trials offering expanded access were more likely to be published (0.71; *p < 0.05*). For COVID-19 studies, use of a crossover intervention model was strongly negatively associated with publication likelihood (−4.56; *p < 0.05*), potentially reflecting challenges in trial design appropriateness or operational feasibility during the early pandemic period.

## Discussion

### Main findings

In this large-scale analysis of over 11,870 interventional trials across CVD, cancer, and COVID-19, we found that stronger oversight, larger operational scale, and greater transparency were consistently linked to a higher probability of publishing and faster publication in peer-reviewed journals. Trials with a DMC, randomization, robust blinding, larger enrollments, more study sites, more secondary outcomes, and posted results were more likely to be published sooner than those without these features. However, the strength and details of these patterns varied by context. In CVD trials, faster publication was linked to DMC presence, randomization, older adult inclusion, and higher masking, while privately funded, supportive care, and diagnostic trials lagged. In cancer, larger enrollment, more sites, expanded access, and result reporting promoted publication, whereas supportive care, basic science, and healthy volunteer trials were slower. For COVID-19, quicker publication was associated with larger enrollment and expanded access, but trials completed later in the pandemic period were published significantly more slowly, suggesting that the alignment of urgency, funding, and infrastructure that characterized the initial pandemic response diminished over time. Subgroup analysis of DMC demonstrates a greater number of secondary outcomes consistently predicted faster publication across CVD, cancer, and COVID-19, while single-arm design in cancer trials was linked to slower publication. In randomized trials, U.S. site involvement and use of an intervention model other than crossover and single-group design were associated with faster publication in CVD. Phases 2 and 3 in cancer studies were more likely to be published, whereas the inclusion of healthy volunteers in CVD was strongly linked to slower dissemination. The results show that even among rigorously designed studies, trial structure, geographic setting, and participant characteristics can meaningfully affect publication. Collectively, these results highlight that operational scale, methodological rigor, and proactive result reporting remain critical levers for shortening the time from trial completion to peer-reviewed evidence. The implications are that trials without these features are less likely to benefit the public good because the results do not make it into the public domain.

### Relation to prior work

Our findings are broadly consistent with prior evidence showing that trial characteristics such as sample size, randomization, and funding source influence the likelihood and timeliness of publication [11,13–17]. Previous analyses have demonstrated that larger trials are generally more likely to be published but can sometimes face delays due to complex enrollment and analysis requirements [18,19]. Randomized controlled trials tend to be more publishable than non-randomized designs, although added complexity can extend timelines [20]. Disease area matters as well; for instance, oncology trials have historically reported lower timely publication rates than other specialties [20,21]. By comparing patterns across CVD, cancer, and COVID-19 trials, our study underscores both common predictors and how exceptional contexts (e.g., the urgency of a pandemic) can accelerate dissemination when scientific and operational momentum align.

### Interpreting headline predictors

Key predictors, including DMC presence, randomization, larger enrollment, more sites, higher masking, and timely result posting, likely reflect broader institutional capacity and trial rigor, not isolated design choices that independently drive faster publication. Well-resourced sponsors and networks are better positioned to implement oversight, recruit diverse populations, measure more outcomes, and manage multi-site operations, all of which support stronger findings and smoother peer review. Thus, simply adding a DMC or masking to a small, under-resourced study may not accelerate publication without broader support. As prior research shows, complete result reporting, enrollment success, and trial phase or scope remain critical to producing publishable, practice-informing evidence.

### Policy implications and the causality caveat

Our findings highlight several actionable areas to reduce publication delays and improve the dissemination of clinical trial evidence. Strengthening independent oversight, particularly through the presence of DMCs, and promoting rigorous design features such as randomization and masking are consistently associated with faster publication. These features likely signal methodological quality and facilitate smoother peer review, suggesting that enhancing trial rigor could accelerate dissemination. Additionally, supporting larger operational scale through adequate funding, multi-site collaboration, and robust infrastructure may help overcome logistical and institutional barriers that hinder timely reporting.

However, while these characteristics are associated with faster publication, the relationships are not necessarily causal. Trials with more rigorous designs and greater scale may benefit from being embedded in better-resourced institutions or sponsor networks with established pipelines for dissemination. These advantages may not be replicable through design choices alone. Indeed, our data show that trials conducted at single sites, lacking U.S. facilities, or funded by private or ambiguous sources were consistently slower to publish, pointing to deeper structural challenges related to infrastructure, visibility, and incentives. For example, industry-funded trials may prioritize regulatory milestones over peer-reviewed publication, while single-site studies may struggle with resource limitations, limited generalizability, or publication gatekeeping.

Therefore, improving publication rates requires more than mandating methodological features; it calls for investments in shared infrastructure, clearer expectations from sponsors, and accountability mechanisms for timely dissemination. Addressing these systemic challenges, particularly for under-resourced trials, is essential to ensure that evidence generation efforts translate into public health benefit. While methodological rigor is an important enabler, its effectiveness ultimately depends on the organizational quality, incentives, and collaborative ecosystems in which trials are conducted.

### Why peer-reviewed publication still matters

Finally, this study reinforces why peer-reviewed journal publication continues to matter, even in an era of mandatory trial registration and result posting. Registries like ClinicalTrials.gov provide a baseline for transparency, but journal articles add critical value by subjecting findings to independent peer review, providing context and detailed analysis, and making results discoverable and citable within the broader evidence base. Publication helps integrate individual trials into systematic reviews, clinical guidelines, and health policy decisions. As our findings show, trials with stronger oversight, broader operational scope, and more rigorous designs were more likely to reach publication, underscoring that registries alone cannot fully substitute for high-quality, peer-reviewed dissemination.

### Limitations and future directions

To link PubMed articles to ClinicalTrials.gov records, we used NCT identifiers as unique tags. While efficient, this approach can undercount publications, as some articles omit NCT numbers. A sensitivity check in CVD trials suggested that ∼19% of “unpublished” studies likely had publications without NCT links, modestly underestimating true rates. However, this nondifferential misclassification likely did not bias observed associations.

Additionally, we recognize that ClinicalTrials.gov may contain incomplete or inconsistently reported information about funding sources, which served as one of our key predictor variables. Prior studies [13,22] have documented substantial inconsistencies in the way sponsor and funding details are reported in the registry, particularly for trials with non-governmental or industry support. These discrepancies may result in the under identification of private or governmental funding in some trials, which could attenuate observed associations between sponsor type and publication outcomes in our analysis. While we attempted to mitigate this issue by disaggregating funding into finer-grained subgroups (e.g., NIH, industry, network), some degree of misclassification is likely, and we now explicitly acknowledge this as a limitation. Future efforts may benefit from triangulating registry data with sponsor disclosures in publications to enhance the accuracy of funding classification.

We also noted that testing multiple predictors across three Cox models may raise the possibility of spurious associations, but our analyses were descriptive rather than confirmatory, so we did not adjust for multiple comparisons. In addition, we modeled each disease area separately rather than using a pooled model, which limited formal interaction testing but allowed inclusion of richer, area-specific covariates given differing missingness patterns. Future work could explore integrated models, improved linkage methods, and targeted interventions to reduce publication delays across trial settings.

## Conclusion

This study highlights the key operational and methodological factors that shape whether and how quickly interventional trials reach publication. Across more than 10,000 CVD, cancer, and COVID-19 trials, timely publication was strongly associated with DMC presence, methodological rigor (randomized and masking) and greater operational scale (large enrollment, multi-site participation, multiple outcome measures). Transparent practices, particularly the posting of trial results, also accelerated dissemination. Conversely, trials with private or other non-governmental funding, single-site settings experienced slower publication. Subgroup analysis showed that even among rigorous designs (DMC and randomized), specific structural and design choices influence dissemination speed and vary by disease. Collectively, these findings underscore the need to strengthen infrastructure, promote rigorous and transparent practices, and address structural barriers to support more equitable and timely dissemination. Our survival analysis reveals actionable opportunities to improve trial design, transparency, and the translation of findings into clinical and policy impact.

## Supporting information

10.1017/cts.2025.10201.sm001 Wang et al. supplementary material Wang et al. supplementary material

## Data Availability

The data that support the findings of this study are available from the author Le Li or Haoyuan Wang upon reasonable request.

## Supplementary material

The supplementary material for this article can be found at https://doi.org/10.1017/cts.2025.10201.

## Code availability statement

The custom code that supports the findings of this study is available from the author Le Li or Haoyuan Wang upon reasonable request. The code is provided for academic, non-commercial use only.
