# Supplementary material for: Determinants of publication likelihood and timeliness for clinical studies
Source: J Clin Transl Sci. 2025 Dec 4;10(1):e1. doi: 10.1017/cts.2025.10201 (PMC12797179; doi:10.1017/cts.2025.10201)
Supplement: Wang et al. supplementary material [file S205986612510201Xsup001.docx]

#

# **Supplementary Materials of**

# **“Determinants of Publication Likelihood and Timeliness for Clinical Studies”**

#

# **Table S1. Variable description**

### **Trial Oversight & Transparency**

| **New Variable Name** | **Description** |
| --- | --- |
| **Trial Oversight & Transparency** | |
| Has DMC | Trial included a Data Monitoring Committee (DMC), which oversees participant safety and trial integrity. |
| Results Reported | Trial posted results on ClinicalTrials.gov. Reflects regulatory compliance and transparency. |
| Expanded Access | Trial provided expanded (compassionate) access to investigational treatments outside the trial. |
| **Study Design Features** | |
| Allocation Randomized | Participants were randomly assigned to different treatment groups. |
| Non-Standard Intervention | Trial used a non-standard intervention model, not falling into parallel, crossover, or single group designs. |
| Crossover Intervention | Participants received treatments in a crossover fashion (e.g., sequence A→B or B→A). |
| Single Group Intervention | All participants received the same treatment without a comparator group. |
| **Trial Phase** | |
| Early Phase | Phase 1 or Phase1/Phase2 |
| Phase 2 | Phase 2 trial, focused on efficacy and side effects. |
| Phase 3 | Phase 3 trial, designed to confirm effectiveness and monitor side effects in larger groups. |
| Phase 4 | Post-marketing study to monitor long-term effectiveness/safety. |
| **Participant Characteristics** | |
| Include Children | Trial included pediatric participants (<18 years). |
| Include Adults | Trial included adults (typically 18–64 years). |
| Include Older Adults | Trial included older adults (≥65 years). |
| Male Only | Trial restricted enrollment to males only. |
| Female Only | Trial restricted enrollment to females only |
| Minimum Age | Minimum eligible age, expressed in days (e.g., 6570 days = 18 years). |
| Healthy Volunteers | Trials were labeled as including healthy volunteers if they explicitly enrolled participants without the target disease. In COVID-19 trials, were primary focus was on vaccine and prophylaxis studies. |
| **Study Purpose** | |
| Primary Purpose Health Services Research | Trial focused on healthcare delivery, cost, access, or utilization. |
| Primary Purpose Basic Science | Trial aimed to understand fundamental biological mechanisms. |
| Primary Purpose Prevention | Trial aimed to prevent disease or adverse outcomes. |
| Primary Purpose Supportive Care | Trial aimed to improve comfort, function, or quality of life (e.g., palliative care). |
| Primary Purpose Diagnostic | Trial tested methods to detect or diagnose a condition |
| Primary Purpose Other | Primary purpose did not fit standard categories. |
| **Blinding (Masking)** | |
| Single Blinding | Only one party (e.g., participants) was blinded. |
| Double Blinding | Two parties (typically participants and investigators) were blinded. |
| Triple Blinding | Three parties (e.g., participant, investigator, assessor) were blinded. |
| Quadruple Blinding | All four parties (participant, care provider, investigator, assessor) were blinded. |
| **Operational Characteristics** | |
| Number of Facilities | Total number of study sites involved in the trial. |
| Has U.S. Facility | At least one site was located in the United States. |
| Single Facility | Trial was conducted at a single location only. |
| Enrollment | Planned or actual number of participants in the trial. |
| Number of Arms | Number of treatment groups (arms) in the trial. |
| **Outcome** | |
| Number of Primary Outcomes to Measure | Number of primary outcomes the trial aimed to evaluate. |
| Number of Secondary Outcomes to Measure | Number of secondary outcomes, which provide additional information. |
| Results Count | The number of result entries or publications linked to the trial. |
| **Funding Source** | |
| Source | Origin of the data entry (e.g., ClinicalTrials.gov or other registry source). |
| Source Class Government | Trials funded by U.S. federal agencies, the National Institutes of Health (NIH), or other government entities |
| Source Class Other | Trials funded by other or unknown sources |
| Source Class Private | Trials funded by individual investigators, industry sponsors (e.g., pharmaceutical or device companies), or research networks |
| **Grouping** | |
| Year Group 1 | Indicates the first categorical year grouping for analysis (year before January 2021) |
| Year Group 2 | Indicates the second categorical year grouping for analysis (year after January 2021)  . |
| **Study Timing & Duration** | |
| Actual Duration | The actual length of time (months) that the clinical trial lasted. |
| Months to Completion Date | Number of months from study registration to actual completion date. |
| Months to Study First Submitted Date | Number of months from start of trial to the date the study was first submitted. |
| Months to Last Updated Submitted Date | Number of months from trial start to last submitted update on the registry. |
| Months to Study First Posted Date | Number of months from study start to when it was first publicly posted. |

# **Appendix A. Definition and Use of Disease-Related Terms to Identify CVD and Cancer Trials**

##

## **CVD and Cancer Cohort**

To construct cohorts of clinical trials related to cardiovascular disease (CVD) and cancer, we relied on a previously established methodology for identifying relevant medical terms, as described by Tasneem et al.[^1^](https://paperpile.com/c/NhEW1c/jcHk). In their study, clinical experts systematically reviewed and annotated disease condition terms submitted to ClinicalTrials.gov for interventional studies registered between October 2007 and September 2010. Both Medical Subject Headings (MeSH) and frequently used non-MeSH (free-text) terms were evaluated for their relevance to three major disease areas: cardiovascular, oncology, and mental health. MeSH terms were drawn from four branches of the 2010 MeSH thesaurus (C, E, F, and G), and only those terms consistently tagged as relevant across all tree locations were retained. Similarly, free-text terms that appeared in five or more interventional studies during the period were also assessed and tagged by domain experts. These curated term lists are available in the AACT database (<https://aact.ctti-clinicaltrials.org/shared_data/proj_tag_study_characteristics>).

In our analysis, we used these curated term lists to identify CVD and cancer studies from a broader set of registered interventional trials in AACT. Specifically, we searched for the presence of these lower-case terms in the conditions field of the AACT database. A study was included in the cardiovascular or cancer cohort if it contained at least one of the relevant lower-case terms. By applying this method, we constructed two disease-specific cohorts for downstream analysis of clinical trial characteristics and publication outcomes.

## **Covid-19 Cohort**

To construct the COVID-19 cohort, we identified interventional clinical trials focused on COVID-19. The first cluster of COVID-19 cases was identified on December 12, 2019, and the World Health Organization officially declared the outbreak a pandemic on March 11, 2020.[^2^](https://paperpile.com/c/NhEW1c/VHwc) Given the rapid emergence of COVID-19 as a global public health crisis, a wide array of clinical trials were registered in response to the pandemic.In our analysis, we identified COVID-19 trials by searching the conditions and browse_conditions fields of the AACT database for relevant disease-specific terms. Only interventional studies were included in our study.

# **Appendix B. Missing Rate Per Variable for CVD, Cancer, Covid-19 cohort**

### **a. Missing Rate per Variable for CVD Cohort**


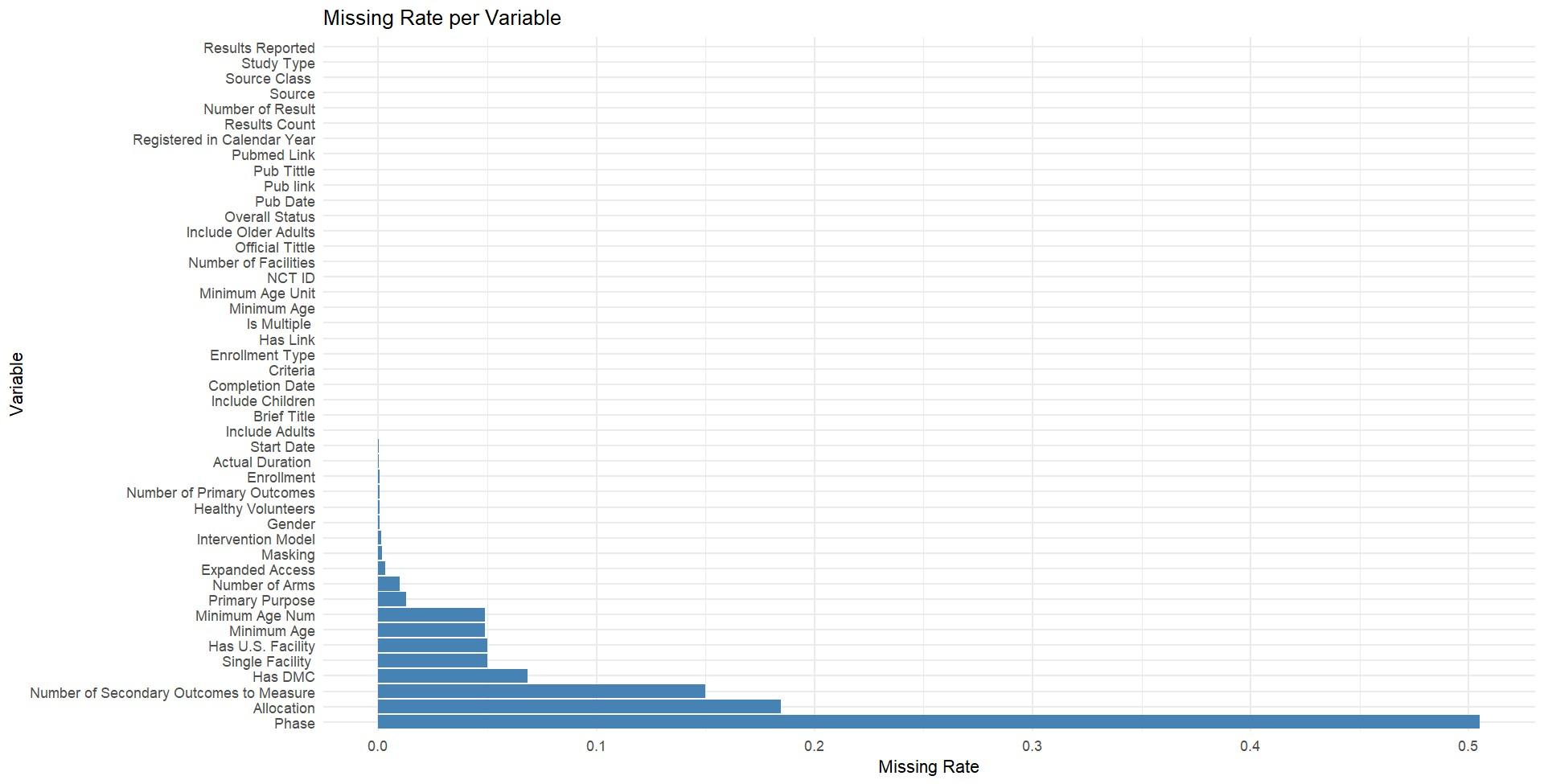


### **b. Missing Rate per Variable for Cancer Cohort**


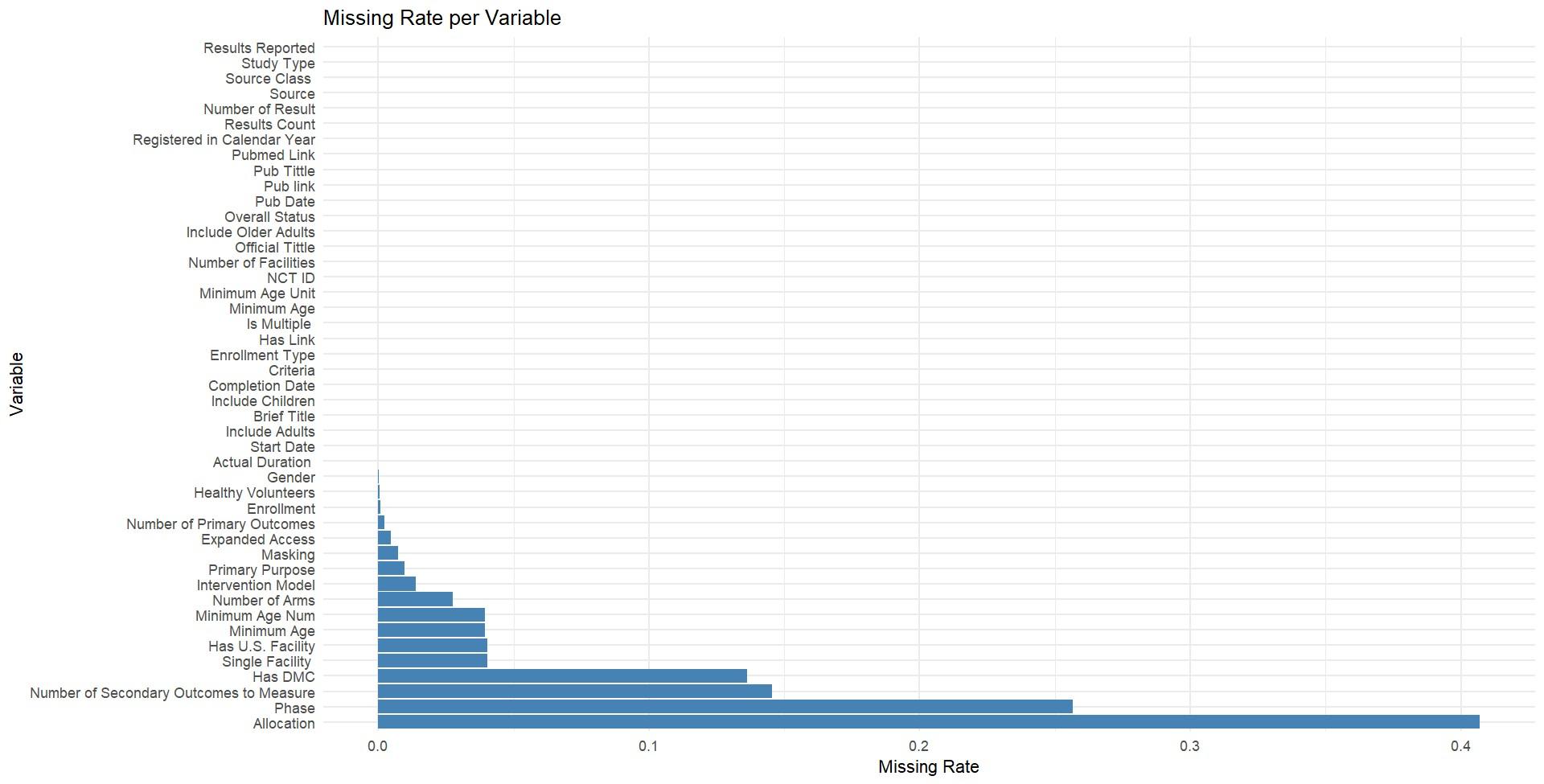


### **c. Missing Rate per Variable for Covid-19 Cohort**

##
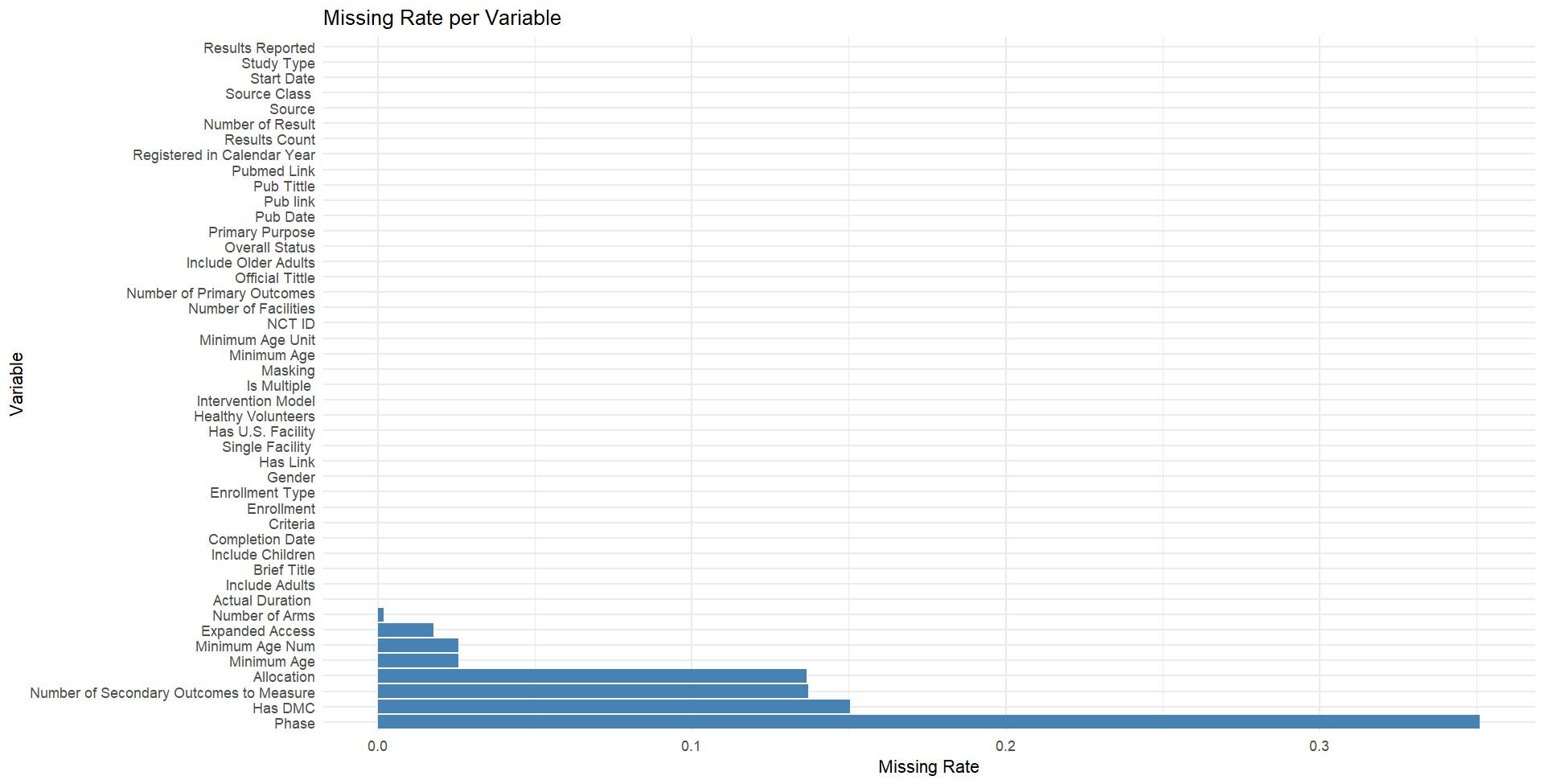


##

# **Table S2. Comparison of study characteristics between clinical trials with and without a linked PubMed publication for trials with U.S. Sponsor**

## **Study characteristics for CVD trials. ( N=2217 )**

| **Variables** | **Category** | **Publication = 0** | **Publication = 1** | **P-value** |
| --- | --- | --- | --- | --- |
| N |  | 1206 | 1011 |  |
| Has DMC, No. (%) | No | 743 ( 61.6) | 452 ( 44.7) | <0.001 |
|  | Yes | 463 ( 38.4) | 559 ( 55.3) |  |
| Source, No. (%) | Government | 55 ( 4.6) | 28 ( 2.8) | 0.007 |
|  | Private | 306 ( 25.4) | 304 ( 30.1) |  |
|  | Other | 845 ( 70.1) | 679 ( 67.2) |  |
| Actual Duration, mean (SD) |  | 30.08 (21.93) | 31.41 (18.60) | 0.126 |
| Results Reported, No. (%) | No | 673 ( 55.8) | 418 ( 41.3) | <0.001 |
|  | Yes | 533 ( 44.2) | 593 ( 58.7) |  |
| Enrollment, mean (SD) |  | 1242.78 (36309.49) | 1480.91 (16067.72) | 0.847 |
| Gender, No. (%) | All | 1144 ( 94.9) | 967 ( 95.6) | 0.684 |
|  | Female | 47 ( 3.9) | 33 ( 3.3) |  |
|  | Male | 15 ( 1.2) | 11 ( 1.1) |  |
| Expanded Access, No. (%) | No | 1203 ( 99.8) | 1007 ( 99.6) | 0.815 |
|  | Yes | 3 ( 0.2) | 4 ( 0.4) |  |
| Number of Facilities, mean (SD) |  | 5.47 (14.85) | 23.42 (80.02) | <0.001 |
| Has U.S. Facility, No. (%) | No | 115 ( 9.5) | 94 ( 9.3) | 0.906 |
|  | Yes | 1091 ( 90.5) | 917 ( 90.7) |  |
| Single Facility, No. (%) | No | 364 ( 30.2) | 468 ( 46.3) | <0.001 |
|  | Yes | 842 ( 69.8) | 543 ( 53.7) |  |
| Primary Purpose, No. (%) | Treatment | 726 ( 60.2) | 630 ( 62.3) | 0.005 |
|  | Supportive Care | 76 ( 6.3) | 51 ( 5.0) |  |
|  | Prevention | 168 ( 13.9) | 161 ( 15.9) |  |
|  | Diagnostic | 42 ( 3.5) | 21 ( 2.1) |  |
|  | Basic Science | 69 ( 5.7) | 30 ( 3.0) |  |
|  | Health Services Research | 54 ( 4.5) | 59 ( 5.8) |  |
|  | Other | 71 ( 5.9) | 59 ( 5.8) |  |
| Number of Arms, mean (SD) |  | 2.44 (1.32) | 2.57 (1.59) | 0.045 |
| Classification of Arms, No. (%) | Number of Arms = 1 | 18 ( 1.5) | 4 ( 0.4) | 0.009 |
|  | Number of Arms = 2 | 908 ( 75.3) | 739 ( 73.1) |  |
|  | Number of Arms > 2 | 280 ( 23.2) | 268 ( 26.5) |  |
| Intervention Model, No. (%) | Parallel | 914 ( 75.8) | 830 ( 82.1) | <0.001 |
|  | Crossover | 154 ( 12.8) | 99 ( 9.8) |  |
|  | Single Group | 97 ( 8.0) | 42 ( 4.2) |  |
|  | Other | 41 ( 3.4) | 40 ( 4.0) |  |
| Allocation, No. (%) | Non Randomized | 179 ( 14.8) | 66 ( 6.5) | <0.001 |
|  | Randomized | 1027 ( 85.2) | 945 ( 93.5) |  |
| Healthy Volunteers, No. (%) | No | 921 ( 76.4) | 845 ( 83.6) | <0.001 |
|  | Yes | 285 ( 23.6) | 166 ( 16.4) |  |
| Months to Completion Date, mean (SD) |  | 30.05 (21.71) | 31.40 (18.42) | 0.12 |
| Months to Study First Submitted Date, mean (SD) |  | 3.36 (16.32) | 0.54 (11.60) | <0.001 |
| Months to Last Updated Submitted Date, mean (SD) |  | 58.57 (34.29) | 62.79 (31.78) | 0.003 |
| Months to Study First Posted Date, mean (SD) |  | 4.48 (17.56) | 1.36 (12.27) | <0.001 |
| Number of Primary Outcomes to Measure, mean (SD) |  | 1.79 (2.27) | 1.79 (2.15) | 0.991 |
| Number of Secondary Outcomes to Measure, mean (SD) |  | 4.51 (5.13) | 6.63 (9.46) | <0.001 |
| Blinding, No. (%) | None | 556 ( 46.1) | 389 ( 38.5) | 0.001 |
|  | Single | 248 ( 20.6) | 223 ( 22.1) |  |
|  | Double | 148 ( 12.3) | 137 ( 13.6) |  |
|  | Triple | 115 ( 9.5) | 98 ( 9.7) |  |
|  | Quadruple | 139 ( 11.5) | 164 ( 16.2) |  |
| Minimum Age, mean (SD) |  | 7872.89 (4042.71) | 7993.64 (4087.12) | 0.486 |
| Include Adults, No. (%) | No | 50 ( 4.1) | 29 ( 2.9) | 0.133 |
|  | Yes | 1156 ( 95.9) | 982 ( 97.1) |  |
| Include Children, No. (%) | No | 1136 ( 94.2) | 961 ( 95.1) | 0.426 |
|  | Yes | 70 ( 5.8) | 50 ( 4.9) |  |
| Include Older Adults, No. (%) | No | 160 ( 13.3) | 84 ( 8.3) | <0.001 |
|  | Yes | 1046 ( 86.7) | 927 ( 91.7) |  |
| Is Multiple , No. (%) | No | 1161 ( 96.3) | 565 ( 55.9) | <0.001 |
|  | Yes | 45 ( 3.7) | 446 ( 44.1) |  |
| Results Count, mean (SD) |  | 0.00 (0.00) | 1.00 (0.00) | <0.001 |
| Enrollment, Median (IQR) |  | 51.5 (95.75) | 120 (283.5) | <0.001 |
| Actual Duration, Median (IQR) |  | 25 (27) | 29 (26) | <0.001 |
| Number of Primary Outcomes to Measure, Median (IQR) |  | 1 (1) | 1 (1) | 0.804 |
| Number of Secondary Outcomes to Measure, Median (IQR) |  | 3 (5) | 4 (6) | <0.001 |

## **Study characteristics for Cancer trials. ( N = 2254)**

| **Variables** | **Category** | **Publication = 0** | **Publication = 1** | **P-value** |
| --- | --- | --- | --- | --- |
| N |  | 1593 | 661 |  |
| Has DMC, No. (%) | No | 691 ( 43.4) | 275 ( 41.6) | 0.467 |
|  | Yes | 902 ( 56.6) | 386 ( 58.4) |  |
| Source, No. (%) | Government | 61 ( 3.8) | 31 ( 4.7) | 0.001 |
|  | Private | 573 ( 36.0) | 288 ( 43.6) |  |
|  | Other | 959 ( 60.2) | 342 ( 51.7) |  |
| Phase, No. (%) | Early Phase | 788 ( 49.5) | 241 ( 36.5) | <0.001 |
|  | Phase 2 | 644 ( 40.4) | 275 ( 41.6) |  |
|  | Phase 3 | 117 ( 7.3) | 126 ( 19.1) |  |
|  | Phase 4 | 44 ( 2.8) | 19 ( 2.9) |  |
| Actual Duration, mean (SD) |  | 44.26 (25.71) | 42.19 (22.82) | 0.073 |
| Results Reported, No. (%) | No | 911 ( 57.2) | 332 ( 50.2) | 0.003 |
|  | Yes | 682 ( 42.8) | 329 ( 49.8) |  |
| Enrollment, mean (SD) |  | 86.15 (225.46) | 181.23 (442.02) | <0.001 |
| Gender, No. (%) | All | 1273 ( 79.9) | 525 ( 79.4) | 0.952 |
|  | Female | 198 ( 12.4) | 83 ( 12.6) |  |
|  | Male | 122 ( 7.7) | 53 ( 8.0) |  |
| Expanded Access, No. (%) | No | 1580 ( 99.2) | 647 ( 97.9) | 0.018 |
|  | Yes | 13 ( 0.8) | 14 ( 2.1) |  |
| Number of Facilities, mean (SD) |  | 11.22 (34.51) | 27.88 (67.75) | <0.001 |
| Has U.S. Facility, No. (%) | No | 511 ( 32.1) | 268 ( 40.5) | <0.001 |
|  | Yes | 1082 ( 67.9) | 393 ( 59.5) |  |
| Single Facility, No. (%) | No | 800 ( 50.2) | 413 ( 62.5) | <0.001 |
|  | Yes | 793 ( 49.8) | 248 ( 37.5) |  |
| Primary Purpose, No. (%) | Treatment | 1369 ( 85.9) | 588 ( 89.0) | 0.006 |
|  | Supportive Care | 60 ( 3.8) | 16 ( 2.4) |  |
|  | Prevention | 46 ( 2.9) | 27 ( 4.1) |  |
|  | Diagnostic | 49 ( 3.1) | 20 ( 3.0) |  |
|  | Basic Science | 30 ( 1.9) | 2 ( 0.3) |  |
|  | Health Services Research | 3 ( 0.2) | 2 ( 0.3) |  |
|  | Other | 36 ( 2.3) | 6 ( 0.9) |  |
| Number of Arms, mean (SD) |  | 1.80 (1.42) | 2.14 (2.23) | <0.001 |
| Classification of Arms, No. (%) | Number of Arms = 1 | 895 ( 56.2) | 278 ( 42.1) | <0.001 |
|  | Number of Arms = 2 | 458 ( 28.8) | 261 ( 39.5) |  |
|  | Number of Arms > 2 | 240 ( 15.1) | 122 ( 18.5) |  |
| Intervention Model, No. (%) | Parallel | 535 ( 33.6) | 312 ( 47.2) | <0.001 |
|  | Crossover | 25 ( 1.6) | 8 ( 1.2) |  |
|  | Single Group | 971 ( 61.0) | 311 ( 47.0) |  |
|  | Other | 62 ( 3.9) | 30 ( 4.5) |  |
| Healthy Volunteers, No. (%) | No | 1543 ( 96.9) | 648 ( 98.0) | 0.163 |
|  | Yes | 50 ( 3.1) | 13 ( 2.0) |  |
| Months to Completion Date, mean (SD) |  | 44.07 (25.38) | 42.04 (22.54) | 0.075 |
| Months to Study First Submitted Date, mean (SD) |  | 1.19 (12.23) | 0.88 (12.91) | 0.596 |
| Months to Last Updated Submitted Date, mean (SD) |  | 80.71 (36.61) | 80.35 (35.26) | 0.832 |
| Months to Study First Posted Date, mean (SD) |  | 1.78 (12.52) | 1.39 (13.07) | 0.505 |
| Number of Primary Outcomes to Measure, mean (SD) |  | 1.57 (1.73) | 1.56 (1.75) | 0.855 |
| Number of Secondary Outcomes to Measure, mean (SD) |  | 4.51 (4.69) | 5.85 (6.19) | <0.001 |
| Blinding, No. (%) | None | 1421 ( 89.2) | 551 ( 83.4) | 0.001 |
|  | Single | 38 ( 2.4) | 16 ( 2.4) |  |
|  | Double | 57 ( 3.6) | 33 ( 5.0) |  |
|  | Triple | 31 ( 1.9) | 20 ( 3.0) |  |
|  | Quadruple | 46 ( 2.9) | 41 ( 6.2) |  |
| Minimum Age, mean (SD) |  | 6989.72 (2824.47) | 7038.15 (3050.83) | 0.717 |
| Include Adults, No. (%) | No | 17 ( 1.1) | 10 ( 1.5) | 0.501 |
|  | Yes | 1576 ( 98.9) | 651 ( 98.5) |  |
| Include Children, No. (%) | No | 1533 ( 96.2) | 638 ( 96.5) | 0.836 |
|  | Yes | 60 ( 3.8) | 23 ( 3.5) |  |
| Include Older Adults, No. (%) | No | 50 ( 3.1) | 17 ( 2.6) | 0.558 |
|  | Yes | 1543 ( 96.9) | 644 ( 97.4) |  |
| Is Multiple , No. (%) | No | 1553 ( 97.5) | 460 ( 69.6) | <0.001 |
|  | Yes | 40 ( 2.5) | 201 ( 30.4) |  |
| Results Count, mean (SD) |  | 0.00 (0.00) | 1.00 (0.00) | <0.001 |
| Enrollment, Median (IQR) |  | 37 (58) | 70 (140) | <0.001 |
| Actual Duration, Median (IQR) |  | 40 (33) | 38 (29) | 0.578 |
| Number of Primary Outcomes to Measure, Median (IQR) |  | 1 (0) | 1 (1) | 0.578 |
| Number of Secondary Outcomes to Measure, Median (IQR) |  | 3 (4) | 4 (5) | <0.001 |

## **Study characteristics for COVID-19 trials. ( N = 192 )**

##

| **Variables** | **Category** | **Publication = 0** | **Publication = 1** | **P-value** |
| --- | --- | --- | --- | --- |
| N |  | 92 | 100 |  |
| Has DMC, No. (%) | No | 44 ( 47.8) | 32 ( 32.0) | 0.036 |
|  | Yes | 48 ( 52.2) | 68 ( 68.0) |  |
| Source, No. (%) | Government | 3 ( 3.3) | 2 ( 2.0) | 0.592 |
|  | Private | 41 ( 44.6) | 39 ( 39.0) |  |
|  | Other | 48 ( 52.2) | 59 ( 59.0) |  |
| Actual Duration, mean (SD) |  | 6.17 (3.90) | 6.97 (4.36) | 0.185 |
| Results Reported, No. (%) | No | 46 ( 50.0) | 25 ( 25.0) | 0.001 |
|  | Yes | 46 ( 50.0) | 75 ( 75.0) |  |
| Enrollment, mean (SD) |  | 5099.47 (33172.07) | 4365.47 (34983.79) | 0.882 |
| Gender, No. (%) | All | 91 ( 98.9) | 97 ( 97.0) | 0.551 |
|  | Female | 0 ( 0.0) | 1 ( 1.0) |  |
|  | Male | 1 ( 1.1) | 2 ( 2.0) |  |
| Expanded Access, No. (%) | No | 92 (100.0) | 97 ( 97.0) | 0.275 |
|  | Yes | 0 ( 0.0) | 3 ( 3.0) |  |
| Number of Facilities, mean (SD) |  | 8.65 (20.52) | 21.82 (41.38) | 0.006 |
| Has U.S. Facility, No. (%) | No | 18 ( 19.6) | 2 ( 2.0) | <0.001 |
|  | Yes | 74 ( 80.4) | 98 ( 98.0) |  |
| Single Facility, No. (%) | No | 39 ( 42.4) | 60 ( 60.0) | 0.022 |
|  | Yes | 53 ( 57.6) | 40 ( 40.0) |  |
| Primary Purpose, No. (%) | Treatment | 67 ( 72.8) | 70 ( 70.0) | 0.742 |
|  | Supportive Care | 2 ( 2.2) | 5 ( 5.0) |  |
|  | Prevention | 12 ( 13.0) | 14 ( 14.0) |  |
|  | Diagnostic | 1 ( 1.1) | 0 ( 0.0) |  |
|  | Basic Science | 1 ( 1.1) | 2 ( 2.0) |  |
|  | Health Services Research | 4 ( 4.3) | 2 ( 2.0) |  |
|  | Other | 5 ( 5.4) | 7 ( 7.0) |  |
| Number of Arms, mean (SD) |  | 2.80 (1.98) | 2.83 (2.00) | 0.929 |
| Classification of Arms, No. (%) | Number of Arms = 2 | 68 ( 73.9) | 74 ( 74.0) | 1 |
|  | Number of Arms > 2 | 24 ( 26.1) | 26 ( 26.0) |  |
| Intervention Model, No. (%) | Parallel | 74 ( 80.4) | 91 ( 91.0) | 0.164 |
|  | Crossover | 3 ( 3.3) | 1 ( 1.0) |  |
|  | Single Group | 4 ( 4.3) | 1 ( 1.0) |  |
|  | Other | 11 ( 12.0) | 7 ( 7.0) |  |
| Allocation, No. (%) | Non Randomized | 13 ( 14.1) | 4 ( 4.0) | 0.027 |
|  | Randomized | 79 ( 85.9) | 96 ( 96.0) |  |
| Healthy Volunteers, No. (%) | No | 69 ( 75.0) | 78 ( 78.0) | 0.749 |
|  | Yes | 23 ( 25.0) | 22 ( 22.0) |  |
| Months to Completion Date, mean (SD) |  | 6.54 (3.86) | 7.34 (4.32) | 0.175 |
| Months to Study First Submitted Date, mean (SD) |  | 1.33 (6.48) | -0.55 (1.64) | 0.006 |
| Months to Last Updated Submitted Date, mean (SD) |  | 21.77 (13.09) | 23.47 (11.97) | 0.349 |
| Months to Study First Posted Date, mean (SD) |  | 1.65 (6.59) | -0.40 (1.67) | 0.003 |
| Number of Primary Outcomes to Measure, mean (SD) |  | 2.11 (1.86) | 2.27 (3.35) | 0.684 |
| Number of Secondary Outcomes to Measure, mean (SD) |  | 7.13 (9.02) | 10.32 (14.45) | 0.071 |
| Blinding, No. (%) | None | 33 ( 35.9) | 30 ( 30.0) | 0.906 |
|  | Single | 8 ( 8.7) | 9 ( 9.0) |  |
|  | Double | 19 ( 20.7) | 20 ( 20.0) |  |
|  | Triple | 12 ( 13.0) | 16 ( 16.0) |  |
|  | Quadruple | 20 ( 21.7) | 25 ( 25.0) |  |
| Minimum Age, mean (SD) |  | 7098.55 (2415.10) | 6618.34 (2329.93) | 0.163 |
| Include Adults, No. (%) | No | 0 ( 0.0) | 1 ( 1.0) | 1 |
|  | Yes | 92 (100.0) | 99 ( 99.0) |  |
| Include Children, No. (%) | No | 91 ( 98.9) | 92 ( 92.0) | 0.055 |
|  | Yes | 1 ( 1.1) | 8 ( 8.0) |  |
| Include Older Adults, No. (%) | No | 7 ( 7.6) | 4 ( 4.0) | 0.445 |
|  | Yes | 85 ( 92.4) | 96 ( 96.0) |  |
| Is Multiple , No. (%) | No | 91 ( 98.9) | 46 ( 46.0) | <0.001 |
|  | Yes | 1 ( 1.1) | 54 ( 54.0) |  |
| Results Count, mean (SD) |  | 0.00 (0.00) | 1.00 (0.00) | <0.001 |
| Year Group, No. (%) | 1 | 86 ( 93.5) | 100 (100.0) | 0.029 |
|  | 2 | 6 ( 6.5) | 0 ( 0.0) |  |
| Enrollment, Median (IQR) |  | 76 (168) | 214 (864) | <0.001 |
| Actual Duration, Median (IQR) |  | 6 (6) | 7 (7.25) | 0.079 |
| Number of Primary Outcomes to Measure, Median (IQR) |  | 1 (1) | 1 (1) | 0.079 |
| Number of Secondary Outcomes to Measure, Median (IQR) |  | 4 (6) | 6 (7) | 0.014 |

##

##

# **Figure S1. Subgroup analysis among trials with U.S. Sponsor: Forest plot of log hazard ratio from Cox PH model for predictors of linked PubMed publication.**

1. **Forest plot for CVD trials.**

**
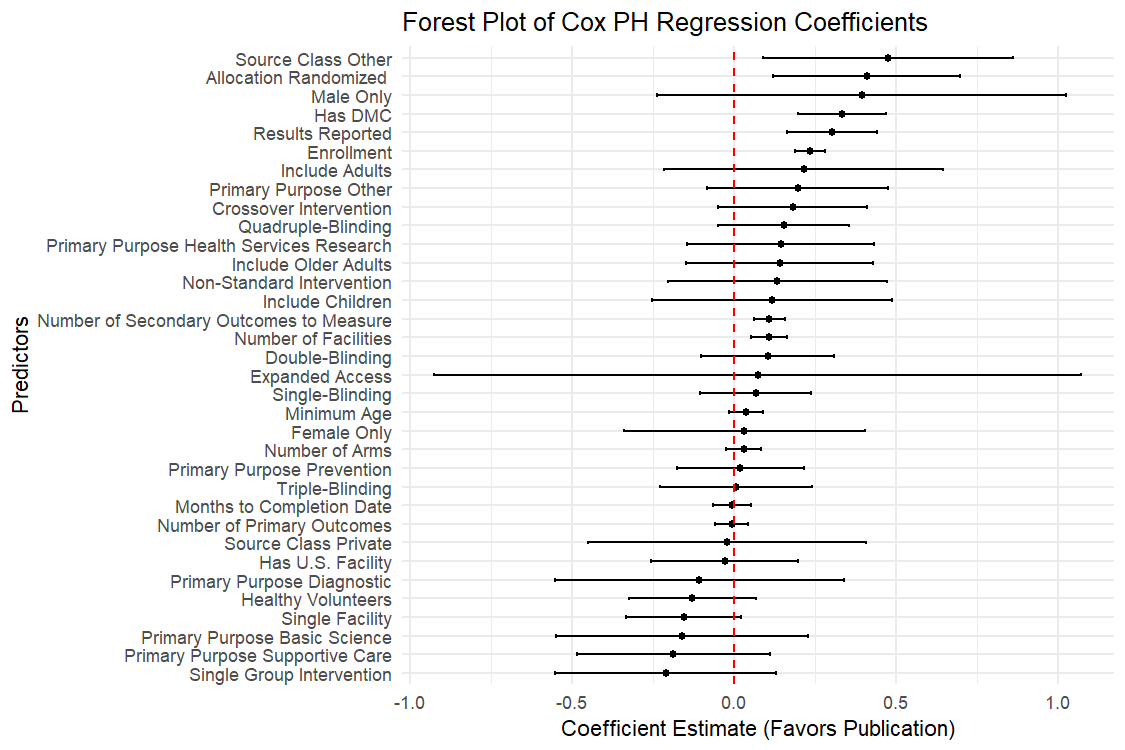
**

1. **Forest plot for Cancer trials.**

**
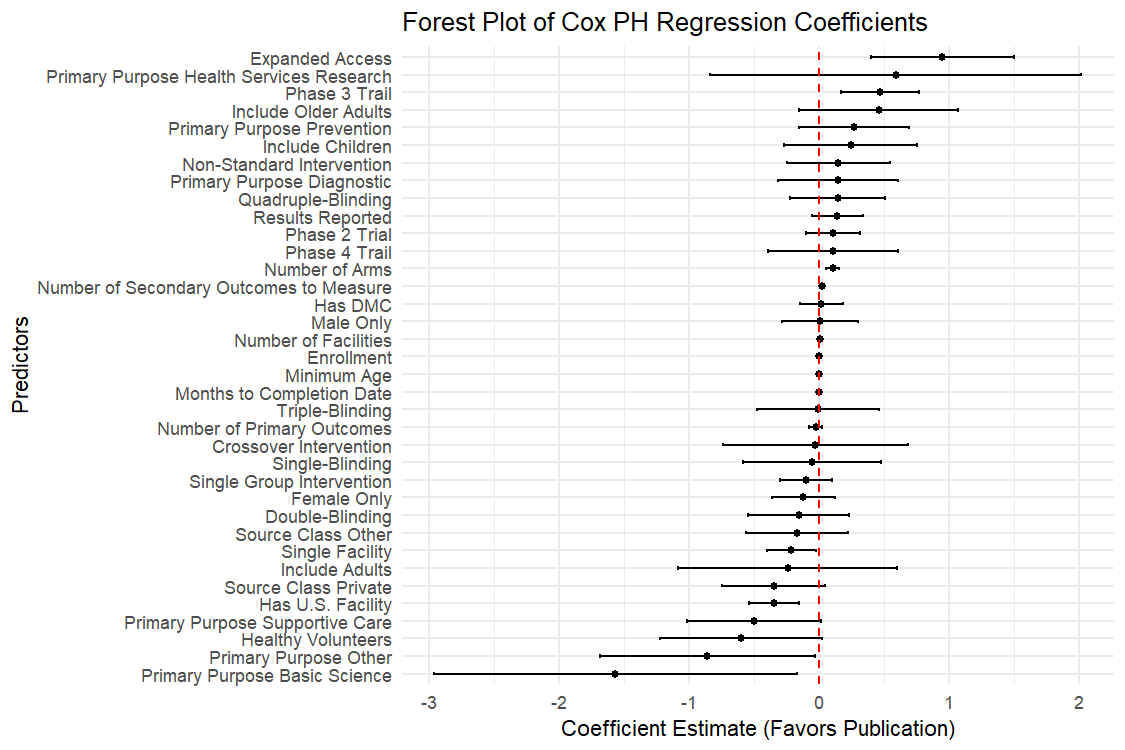
**

1. **Forest plot for COVID-19 trials.**

**
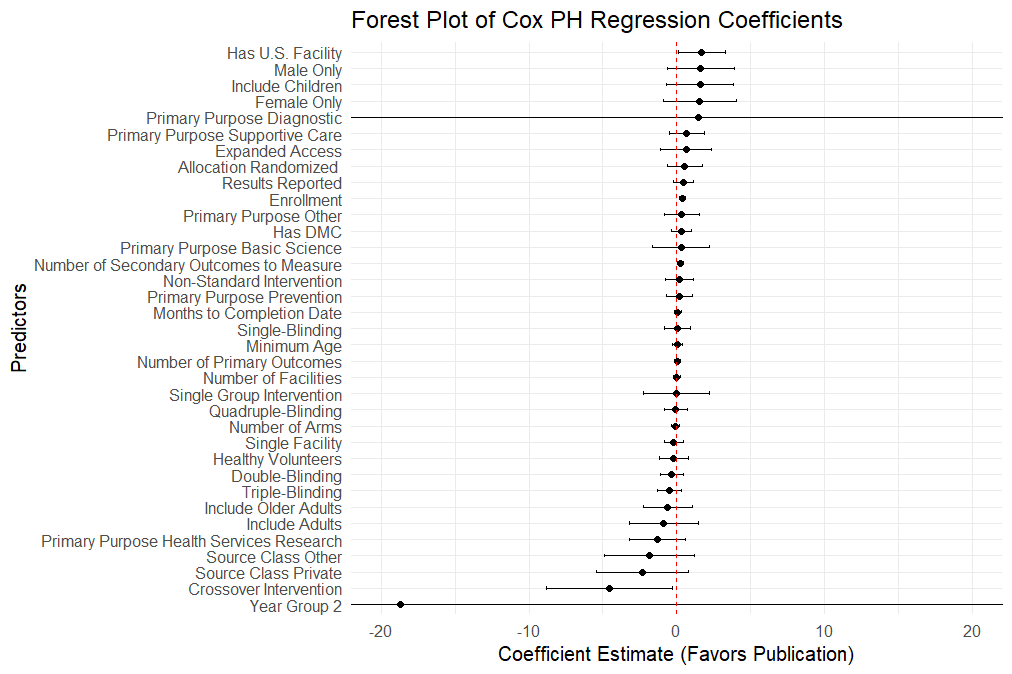
**

##

# **REFERENCES**

1. [Tasneem A, Aberle L, Ananth H, et al. The database for aggregate analysis of ClinicalTrials.gov (AACT) and subsequent regrouping by clinical specialty. *PloS one*. 2012;7(3). doi:](http://paperpile.com/b/NhEW1c/jcHk)[10.1371/journal.pone.0033677](http://dx.doi.org/10.1371/journal.pone.0033677)

2. [CDC. CDC Museum COVID-19 Timeline. Centers for Disease Control and Prevention. January 31, 2025. Accessed July 14, 2025.](http://paperpile.com/b/NhEW1c/VHwc) <https://www.cdc.gov/museum/timeline/covid19.html>
